# The Candidate Splicing Factor Sfswap Regulates Growth and Patterning of Inner Ear Sensory Organs

**DOI:** 10.1371/journal.pgen.1004055

**Published:** 2014-01-02

**Authors:** Yalda Moayedi, Martin L. Basch, Natasha L. Pacheco, Simon S. Gao, Rosalie Wang, Wilbur Harrison, Ningna Xiao, John S. Oghalai, Paul A. Overbeek, Graeme Mardon, Andrew K. Groves

**Affiliations:** 1 Department of Neuroscience, Baylor College of Medicine, Houston, Texas, United States of America; 2 Department of Pathology, Baylor College of Medicine, Houston, Texas, United States of America; 3 Department of Otolaryngology, Baylor College of Medicine, Houston, Texas, United States of America; 4 Department of Bioengineering, Rice University, Houston, Texas, United States of America; 5 Department of Otolaryngology, Stanford University School of Medicine, Palo Alto, California, United States; 6 Department of Molecular and Cellular Biology, Baylor College of Medicine, Houston, Texas, United States of America; 7 Program in Developmental Biology, Baylor College of Medicine, Houston, Texas, United States of America; 8 Department of Molecular and Human Genetics, Baylor College of Medicine, Houston, Texas, United States of America; NIH/NIDCD, United States of America

## Abstract

The Notch signaling pathway is thought to regulate multiple stages of inner ear development. Mutations in the Notch signaling pathway cause disruptions in the number and arrangement of hair cells and supporting cells in sensory regions of the ear. In this study we identify an insertional mutation in the mouse *Sfswap* gene, a putative splicing factor, that results in mice with vestibular and cochlear defects that are consistent with disrupted Notch signaling. Homozygous *Sfswap* mutants display hyperactivity and circling behavior consistent with vestibular defects, and significantly impaired hearing. The cochlea of newborn *Sfswap* mutant mice shows a significant reduction in outer hair cells and supporting cells and ectopic inner hair cells. This phenotype most closely resembles that seen in hypomorphic alleles of the Notch ligand *Jagged1* (*Jag1*). We show that *Jag1; Sfswap* compound mutants have inner ear defects that are more severe than expected from simple additive effects of the single mutants, indicating a genetic interaction between *Sfswap* and *Jag1*. In addition, expression of genes involved in Notch signaling in the inner ear are reduced in *Sfswap* mutants. There is increased interest in how splicing affects inner ear development and function. Our work is one of the first studies to suggest that a putative splicing factor has specific effects on Notch signaling pathway members and inner ear development.

## Author Summary

The organ of Corti is a sensory structure in the cochlea that mediates our sense of hearing. It consists of one row of inner hair cells and three rows of outer hair cells, together with an array of neighboring supporting cells. The precise arrangement of these different cell types is regulated very tightly by a number of signaling pathways during embryonic development, and mutations in genes that regulate this pattern often lead to deafness. We have generated a mouse mutant containing a lentiviral insertion in a gene encoding a putative RNA splicing factor called Sfswap. Homozygous mutant mice have hearing and balance defects, and have an abnormal arrangement of hair cells in their cochlea. These defects are consistent with defects in the Notch signaling pathway. We show that *Sfswap* mutants interact genetically with a mutation in *Jagged1*, which encodes a Notch ligand. We show that expression of some genes involved in Notch signaling is disrupted in *Sfswap* mutant mice. Our work is one of the first studies to show that a putative splicing factor has specific effects on Notch signaling pathway members and on inner ear development.

## Introduction

The organ of Corti is an excellent system to study mechanisms of cell patterning due to its highly organized array of sensory cells. It contains one row of inner hair cells, three rows of outer hair cells and several classes of specialized supporting cells, including pillar and Deiters' cells. The signals responsible for this intricate and fine-grained cellular pattern are beginning to be understood, and include the Notch signaling pathway. The Notch1 receptor is expressed in supporting cells, while the Notch ligands Jagged2 (Jag2), Delta1 and Delta3 are expressed in hair cells after they differentiate from prosensory precursors [1], [2], [3], [4]. Supernumerary inner and outer hair cells are generated at the expense of supporting cells in the absence of *Notch1, Jag2* or *Delta1* [5], [6], [7], [8], [9]. In addition, mutations in members of the *Hes* and *Hey* family of downstream *Notch* effectors also cause an increase in hair cell numbers at the expense of supporting cells, with mutations of multiple *Hes/Hey* family members causing progressively more severe phenotypes [10], [11]. These studies suggest that lateral inhibition mediated by Notch signaling acts to regulate and maintain the correct proportion of hair cells and supporting cells in inner ear sensory organs.

The Notch ligand, Jagged1 (*Jag1*) is expressed in all sensory organs of the inner ear prior to the onset of hair cell differentiation [2], [3]. In the developing mouse cochlea, *Jag1* is expressed broadly at first, and then becomes excluded from the prosensory domain and restricted to Kölliker's organ by E13.5 [12]. As prosensory progenitors in the cochlea differentiate into hair cells and supporting cells, *Jag1* is down-regulated from Kölliker's organ and is expressed with *Notch1* in supporting cells [2], [3]. Although several hypotheses have been proposed for the mechanism of Jag1 function in the developing cochlea, the precise role of this gene is still poorly understood. Conditional inactivation of *Jag1* in the developing inner ear leads to a severely disrupted organ of Corti [6], [13]. Sensory cells are entirely absent from the basal region of the *Jag1* conditional mutant cochlea, whereas two rows of inner hair cells but no outer hair cells are observed in the apical region of the cochlea [6], [13]. *Jag1* mutant heterozygotes generated by ENU mutagenesis show a milder phenotype; they lack some cells in the third row of outer hair cells and display ectopic inner hair cells [14], [15].

As part of a study to determine whether self-inactivating (SIN) lentiviruses can be used for efficient insertional mutagenesis in transgenic mice, we used a tyrosinase-expressing lentiviral vector to infect pre-implantation albino (FVB/N) mouse embryos by subzonal injection. Tyrosinase expression rescues albinism and provides a visible, dosage-sensitive, reporter for different integration sites. Transgenic founder (F0) mice were bred to establish families with single lentiviral integration sites and the mice were then inbred and assayed for evidence of insertional mutations. In one family (OVE2267B), homozygous mic*e* displayed a robust circling behavior, suggesting inner ear defects. The lentiviral integration site in this family was mapped to the *Sfswap* gene. *Sfswap* was originally identified in *Drosophila* as a suppressor of the transposon-induced *white-apricot* mutation [16]. *Sfswap* encodes an RS-domain containing (SR-Like) protein that is a putative splicing factor. RS-domain containing proteins are known to regulate many aspects of RNA processing, including splicing, transcript elongation, transcript stability, nuclear export and miRNA cleavage as well as genome stability, (reviewed in [17]). In *Drosophila*, Sfswap regulates splicing of several genes, including *Sfswap* itself [18], [19], [20]. *In vitro* evidence suggests that Sfswap is involved in RNA processing in mammals as well by promoting fully spliced transcripts [21], [22], [23]. It is unclear whether Sfswap regulates other aspects of RNA processing, however some evidence in *Drosophila* suggests Sfswap may influence transcript stability [18], [24]. Our *Sfswap* mouse mutants have hearing loss, circling behavior, and show cochlear defects that are remarkably similar to those seen in *Jag1* hypomorphic mutants - they show reduced numbers of outer hair cells and their associated supporting cells and increased numbers of inner hair cells. Compound mutants of *Sfswap* and *Jag1* have a more pronounced cochlear phenotype and have truncations of their semicircular canals, suggestive of a genetic interaction. Moreover, we show that levels of expression of a number of genes involved in Notch signaling in the inner ear, such as *Hey1*, *Neurl1, Numb, and MamlD1*, are also affected in the *Sfswap* mutants. Our results suggest that *Sfswap* is necessary for the proper development and patterning of sensory structures of the inner ear and shows a genetic interaction with *Jagged1* which may be mediated by a reduction in several genes involved in Notch signaling.

## Results

### Generation and identification of the *Sfswap^Tg^* allele

**Figure 1:**
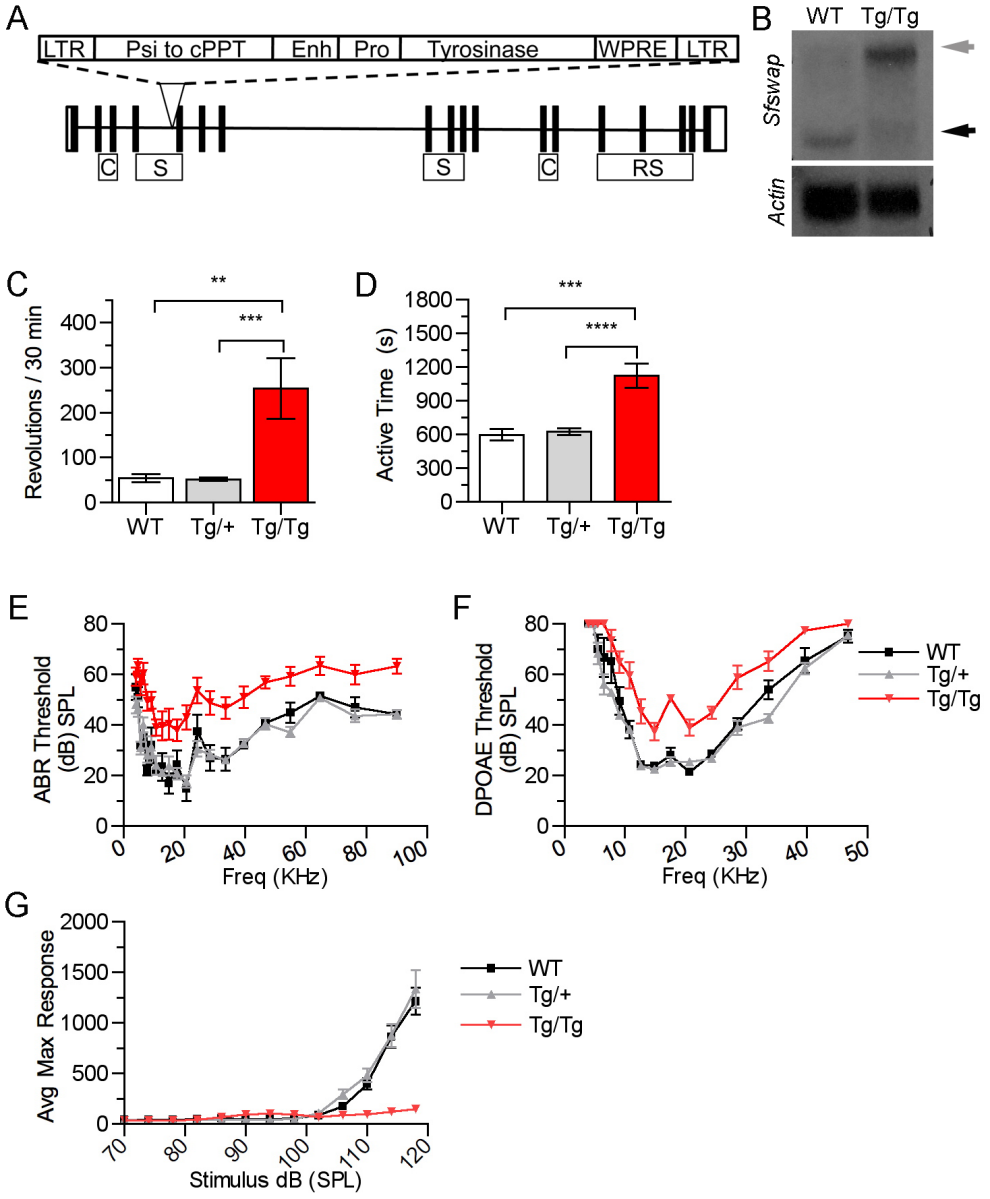
Mutation of *Sfswap* results in mice with vestibular and cochlear defects. (A): Lentiviral integration into the 4^th^ intron of *Sfswap*. The exons of *Sfswap* are shown with black vertical bars. The features of the lentivirus are indicated as follows: LTR: long terminal repeat, Psi: packaging sequence, cPPT: central polypurine tract, Enh: tyrosinase enhancer, Pro: tyrosinase promoter, WPRE: woodchuck hepatitis virus post-transcriptional regulatory element. Features of the *Sfswap* gene are indicated as follows: C: Coil-Coil, S: SURP domain, RS: Arginine-Serine domain. (B): Northern blot of brain RNA from wild-type and *Sfswap^Tg^* mice. The mutation caused by the insertion of the lentivirus results in a reduction of wild-type *Sfswap* transcript (black arrow) and the appearance of a new isoform greater than 10 Kb in size (grey arrow). (C, D): In a 30-minute open field task, *Sfswap^Tg^* mice exhibit increased circling behavior (C) and hyperactivity (D). (E, F): *Sfswap^Tg/Tg^* mice exhibit increased auditory thresholds measured by ABR (E; p(WT, Tg/Tg) = 0.003, p(Tg/+, Tg/Tg) = 2×10^−4^) (black - WT, grey - *Tg/+*, red - *Tg/Tg*) and increased DPOAE thresholds (F; p(WT, Tg/Tg) = 1×10^−4^, p(Tg/+, Tg/Tg) = 3×10^−6^), indicating a hearing deficit. (G): *Sfswap^Tg/Tg^* mice show a reduced startle response. Mice were allowed to acclimate to a 70 dB background noise, and were then exposed to noise of increasing sound pressures. Wild type and *Sfwap ^Tg/+^* mice begin to show a response at 100 dB, whereas *Sfswap^Tg/Tg^* mice do not begin to show a startle response until 118 dB (p(WT, Tg/Tg) = 2.2×10^−7^, p(Tg/+, Tg/Tg) = 1.8×10^−7^).

We conducted a random insertional mutagenesis study using a tyrosinase-tagged lentiviral vector (Figure 1A) to infect pre-implantation albino (FVB/N) mouse embryos. Lentiviral infection provides a number of distinct advantages for insertional mutagenesis. Lentiviral integration sites are scattered throughout the genome, are single copy, and are well-defined since integration is catalyzed by the lentiviral integrase. Since the injection needle is not inserted into the pronucleus of the embryos, the genomic DNA is not mechanically damaged and the yield of transgenic newborns is much higher. The tyrosinase minigene rescues albinism and provides a dosage-dependent, visible, reporter gene. Greater than 85% of the newborn mice from infected embryos were pigmented, verifying efficient transgenesis and effective expression of the reporter gene (data not shown). F0 mice were bred to FVB/N partners to generate F1 offspring and the F1 mice were again bred to albino mice to establish families with a single lentiviral insertion site. Mice were then inbred and assayed for evidence of insertional mutations. Eighty unique mutant phenotypes were identified (data not shown). This manuscript describes the characterization of the insertional mutation in family OVE2267B. Using inverse PCR, the integration site in this family was amplified, sequenced, and shown to be located in the fourth intron of *Sfswap*, 115 bases 5′ of exon 5. The mutation was labeled *Sfswap^Tg(Tyr)2267BOve^* (MGI ID: 5287267), and will be referred to throughout this study as *Sfswap^Tg^* or *Tg*. The tyrosinase minigene allows for identification of genotype by coat color when transgenics are maintained in an albino background [25]. Homozygote *Tg* mice are more darkly pigmented than heterozygotes and non-transgenic mice are albino. We back-crossed *Sfswap^Tg^* mice onto the FVB/N background for more than 10 generations. Tyrosinase expression and the inner ear phenotypes in homozygous mutants correlated 100% with the insertion in *Sfswap* as assayed by PCR. Additionally, Southern blots showed a single lentiviral integration site (data not shown). Northern blot analysis of *Sfswap* revealed that the wild-type *Sfswap* transcript was significantly reduced in *Sfswap^Tg/Tg^* mice (Figure 1B). In its place we observed an accumulation of *Sfswap* RNA migrating at an abnormal size (greater than 10 Kb). This RNA is most likely unspliced or incompletely spliced *Sfswap* mRNA, suggesting a hypomorphic allele. Reverse transcriptase PCR (RT-PCR), followed by sequencing, reveals that some of the *Sfswap* mRNA produced in *Sfswap^Tg/Tg^* mice includes sequences from the lentiviral insert and exclusion of exons surrounding the insert (data not shown). These abnormal RNAs are not found in mice heterozygous for the transgene, suggesting that *Sfswap^Tg^* is likely to be a recessive allele. Previous studies have demonstrated that *Sfswap* can regulate the splicing of its own transcript [18], [21], [22]. Disruption of Sfswap function by the mutation may explain why the *Sfswap* transcript is aberrantly spliced in homozygotes but not heterozygotes.

We initially identified *Sfswap^Tg/Tg^* mutants in our screen because they displayed a significant circling behavior (Figure 1C, Video S1). In a 30-minute open field assay, *Tg/Tg* mice circle almost five times more than wild-type or heterozygous littermates. Sfswap*^Tg/Tg^* mice are also almost twice as active as littermates (Figure 1D). Since circling behavior is often associated with vestibular dysfunction, we tested *Sfswap^Tg/Tg^* mice for balance defects. We found that *Tg/Tg* mice curl and grasp their feet in a tail hang assay, circle and tumble in a forced swim test, and have a delay in righting behavior (data not shown), all indicative of vestibular defects [26]. *Sfswap^Tg/Tg^* mutants on the FVB/N background are about 23% smaller than wild-type (WT) littermates at 8 weeks (WT = 29+/−1.11 g, *Tg/Tg* = 22.28+/−0.99 g p = 0.001), and this size difference is detectable at birth (WT = 1.39+/−0.045 g, *Tg/Tg* = 1.22+/−0.056 g, p = 0.008). *Tg/Tg* animals mate at very low frequency in the FVB background, but when crossed to a C57Bl/6 background, the circling behavior ceases and homozygous mice are able to mate more successfully.

Balance defects are often associated with hearing deficits in mice. To test if *Sfswap* mutants also have hearing defects we measured auditory-evoked brainstem responses (ABR) and found that *Tg/Tg* mice have an average 18 dB increase in threshold to elicit an auditory response, indicating a moderate degree of hearing loss (Figure 1E). Furthermore, ABR waveforms to auditory stimuli presented at intensities above threshold in *Sfswap^Tg/Tg^* mice have a qualitatively normal shape but smaller peak-to-peak amplitudes (Figure S1). This suggests that the auditory pathway is intact in *Sfswap* mice, but that fewer neurons are being stimulated to suprathreshold stimuli compared to wild-type mice. We next performed distortion product otoacoustic emissions assays (DPOAE) to identify if the hearing loss is at the level of hair cells. *Tg/Tg* mutants display an average 15 dB increase in DPOAE thresholds (Figure 1F), suggesting that outer hair cell defects may contribute to the hearing deficits [27]. To test how hearing loss affects behavior, we tested auditory startle responses. In this assay, mice are first acclimated to 70 dB white noise and their movement in response to subsequent varying sound pressures is recorded. As previously reported [28], the magnitude of the startle response increases rapidly between 100–120 dB in wild type mice (Figure 1G). We found that the startle responses in *Tg/Tg* mice are significantly attenuated compared to wild type. The maximum response we observe in *Tg/Tg* mice at 118 dB resembles those seen in wild type at sound pressures between 102–106 dB. This reduction in the threshold required to elicit a startle response resembles the increases we observed in ABR and DPOAE thresholds.

**Figure 2:**
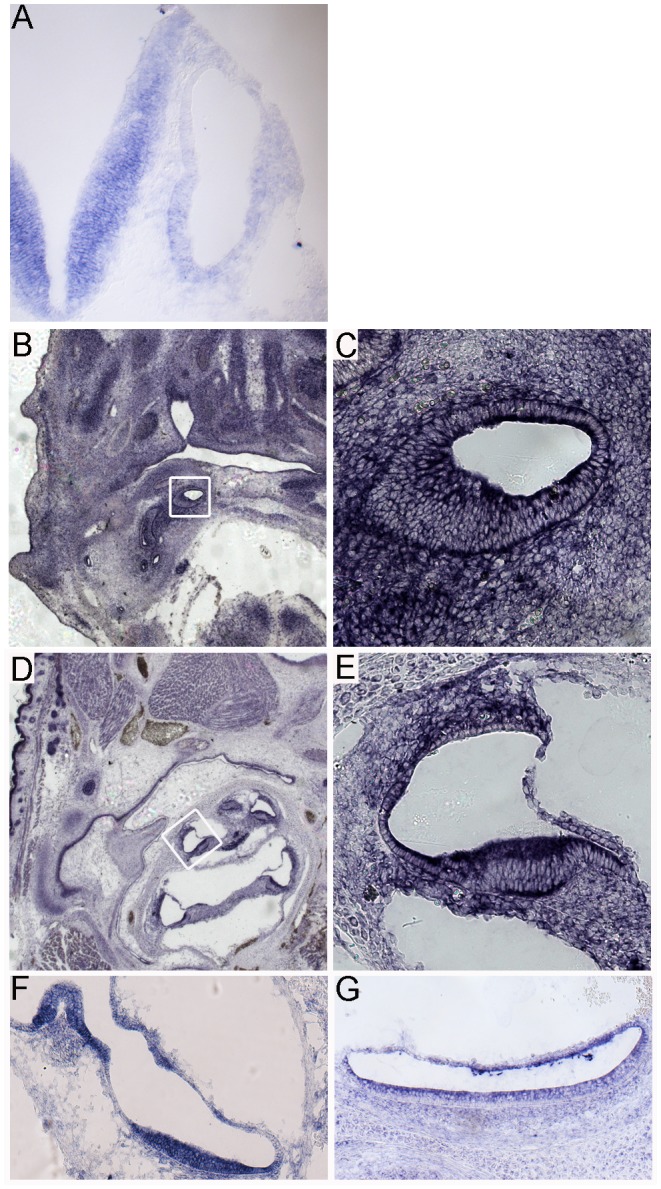
*Sfswap* is expressed in the developing inner ear. (A): *Sfswap* is expressed in the E10.5 inner ear and hindbrain. (B, C): At E13.5, *Sfswap* RNA is expressed broadly throughout the wild-type cochlea and surrounding mesenchyme. The white box in (B) is shown at higher magnification in (C). (D, E): At P0, *Sfswap* expression is more restricted to the wild-type cochlea and the spiral ganglion within the inner ear and is expressed less strongly in surrounding tissues. Note that *Sfswap* is also expressed strongly in the hair follicles and dermis of neonatal mice (D). (F, G): *Sfswap* is expressed in the P0 cristae (F) and maculae of the utricle (F) and saccule (G).

RNA *in situ* hybridization reveals that *Sfswap* is expressed in the inner ear and brain at E10.5 (Figure 2A). *Sfswap* is expressed broadly at low levels throughout the E10.5 embryo. *Sfswap* is expressed broadly and uniformly in the cochlea and surrounding mesenchyme as early as E13.5 in wild-type embryos (Figure 2B, C). Expression of *Sfswap* persists through birth and is maintained broadly in the cochlea, spiral ganglion, cristae, utricle, and saccule, but is reduced in the surrounding tissues (Figure 2D, E, F, G).

### *Sfswap^Tg/Tg^* mutants exhibit defects in hair cells and supporting cells in the cochlea

**Figure 3:**
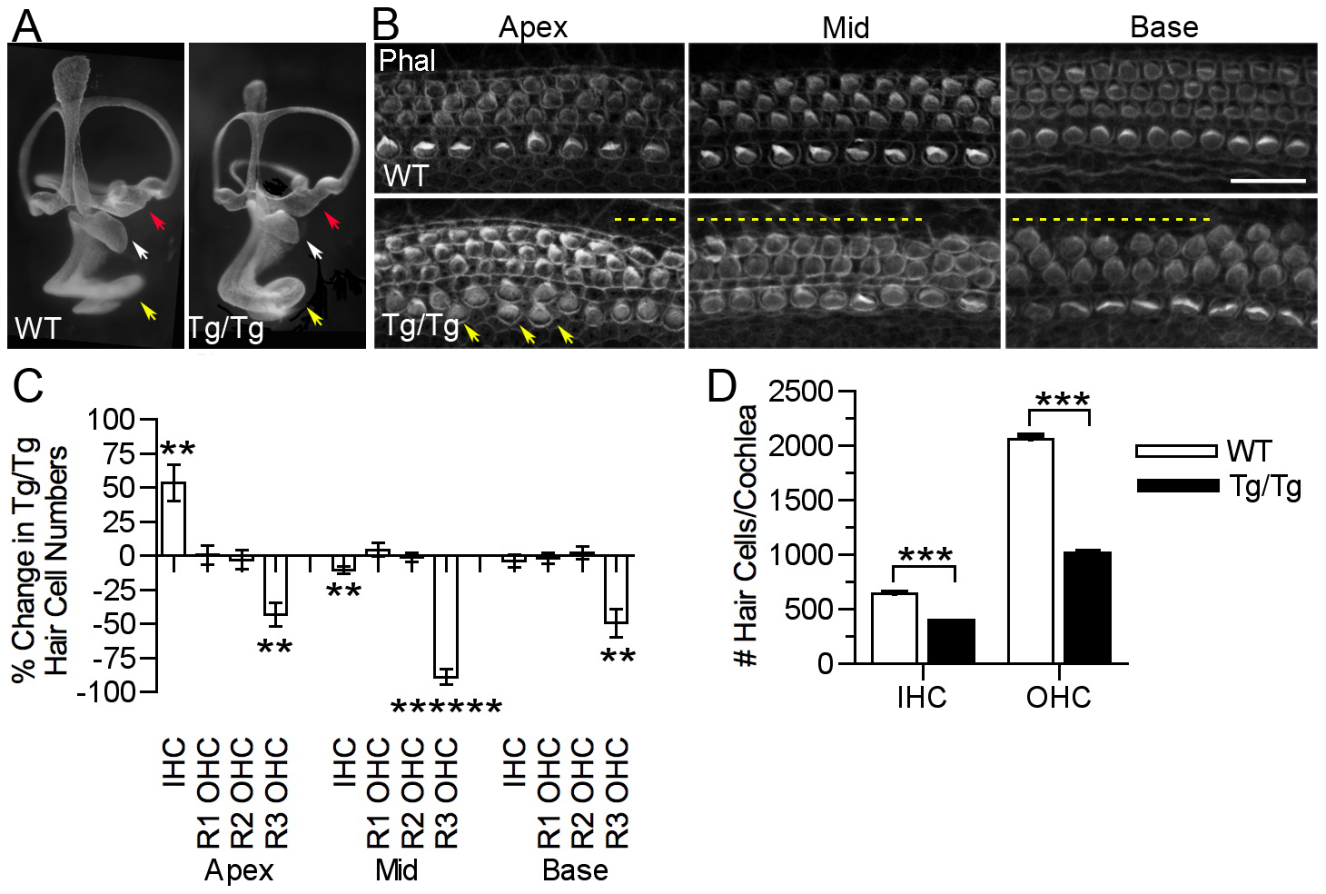
Mutation of *Sfswap* results in a shorter cochlea, fewer outer hair cells and ectopic inner hair cells. (A): Paint fills of E15.5 wild-type and *Sfswap^Tg/Tg^* inner ears. The main components of the inner ear labyrinth are all present, although the utricle (red arrow) and saccule (white arrow) appear smaller and the cochlea (yellow arrow) is reduced in length. (B): *Sfswap^Tg/Tg^* mice are missing hair cells in the third row of outer hair cells (dotted lines) in the basal and mid-turn regions of the cochlea. They also have extra inner hair cells near the apex (arrows). Scale bar = 20 µm. Phal: Phalloidin (C): The distribution of inner and outer hair cells is shown for the apical, mid-turn and basal thirds of the cochlea at P0. Hair cells were counted in 200 µm lengths. The change in hair cell numbers in *Sfswap^Tg/Tg^* mice is shown compared to wild-type controls. Significance is indicated as asterisks and given in Table 1 ** : p≤10^−3^, *** : p≤10^−4^, ****** : p≤10^−7^. (D): Total inner and outer hair cell counts for *Sfswap^Tg/Tg^* mice and wild-type controls (see also Table 1). The decrease in total cell counts reflects the decrease in the length of the mutant cochlea.

To study the effects of the *Sfswap* ^Tg^ mutation on inner ear morphology, we performed paint fills of the inner ear at E13.5–16.5. We found no defects in semicircular canal structure, and all components of the inner ear appear to be present. However, the cochlea is shorter, and the organs of the vestibular labyrinths are reduced in size (Figure 3A). Measurement of the flat-mounted cochlear preparations reveals a 38% reduction in the length of the cochlea (WT = 5058.5+/−201.45 µm *Tg/Tg* = 3135.9+/−221.92 µm p = 5×10^−6^). We stained surface preparations of newborn *Tg/Tg* cochleas with fluorescently-labeled phalloidin to detect actin in stereocilia and found regions of the mutant cochlea are missing the third row of outer hair cells and regions that have ectopic inner hair cells (Figure 3B, C). We quantified the number of hair cells per 200 µm and found there are significantly fewer third row outer hair cells throughout the length of the cochlea and significantly more inner hair cells at the apex (Figure 3C, Table 1). Although inner hair cells are locally increased, we found a significant decrease in total numbers of both inner and outer hair cells (37.5% and 50% respectively; Figure 3D).

**Table 1:** Mutation in Sfswap results in fewer hair cells.

Cochlear Region	Row	WT Count	*Tg/Tg* Count	P value
**Apex**	IHC	24.2+/−0.49	37.2+/−3.10	0.003
	Row1 OHC	27.4+/−0.51	27.6+/−1.78	NS
	Row2 OHC	28.4+/−0.51	27.6+/−1.91	NS
	Row3 OHC	28.8+/−0.37	16.4+/−2.54	0.0013
**Middle**	IHC	24.8+/−0.37	22.2+/−0.58	0.006
	Row1 OHC	26.0+/−0.95	27.2+/−0.86	NS
	Row2 OHC	26.2+/−0.66	26.0+/−0.55	NS
	Row3 OHC	26.6+/−0.75	3.0+/−1.48	5.9E-7
**Base**	IHC	22.4+/−0.68	21.6+/−0.81	NS
	Row1 OHC	24.5+/−0.65	25+/−0.58	NS
	Row2 OHC	25.2+/−0.80	25.8+/−0.80	NS
	Row3 OHC	25.6+/−1.17	13.0+/−2.53	0.0088
**Whole Cochlea**	IHC	644+/−10.5	402+/−5.03	1.6E-4
	OHC	2066+/−29	1021+/−26.2	1.2E-4

The number of hair cells in each row per 200 µm was collected at the apex, mid and base in P0 cochleas. In *Sfswap^Tg/Tg^* mutants, the third row of outer hair cells is reduced in all areas. Loss is greatest in the middle cochlea. The number of inner hair cells is significantly increased at the apex. The total number of hair cells was counted in WT and *Tg/Tg* cochleas at P2 and mutants show a significant decrease in both inner and outer hair cells.

**Figure 4:**
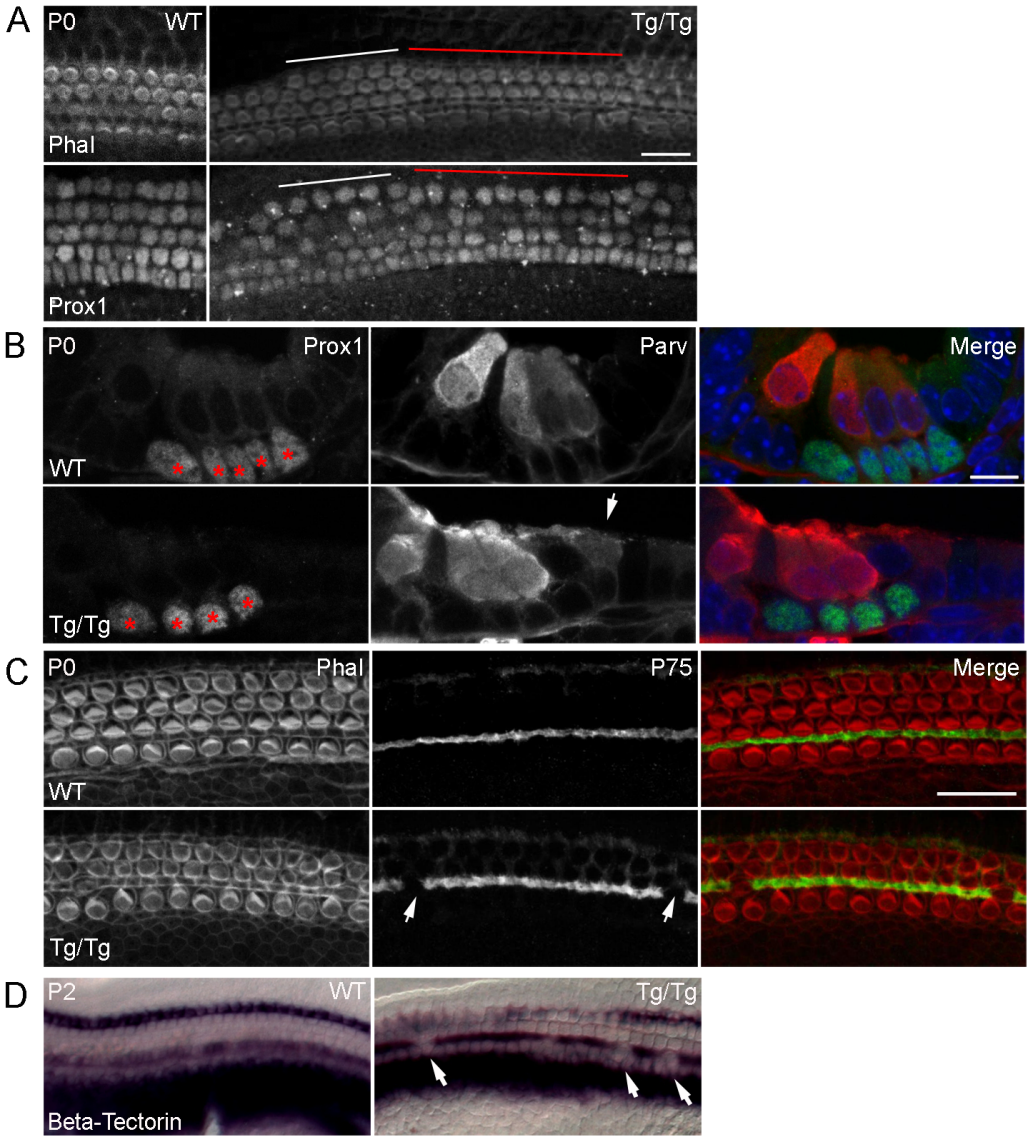
Mutation of *Sfswap* leads to fewer supporting cells. (A): Cochleas from P0 *Sfswap^Tg/Tg^* and wild-type mice co-stained with Prox1 to show supporting cells and with fluorescently-labeled phalloidin (Phal) to reveal hair cells. In areas where hair cells are missing, supporting cells are also missing (white line compared to red line). Scale bar = 20 µm (B): Sections of P0 *Sfswap^Tg/Tg^* and wild-type mice co-stained with Prox1 (green) to show supporting cells and parvalbumin (Parv, red) to show hair cells. Missing hair cells are accompanied by missing supporting cells (asterisks). In addition, ectopic parvalbumin-expressing cells are observed in *Sfswap^Tg/Tg^* mutants (arrow). Scale bar = 10 µm (C, D): Assays for the pillar cell markers p75 (red) at P0 and *β-tectorin* (purple) at P2 shows occasional loss of pillar cells in *Sfswap^Tg/Tg^* mutants. Scale bar = 20 µm.

To determine if there are also defects in supporting cells in *Sfswap^Tg/Tg^* cochleas, we examined expression of the transcription factor Prox1 on whole mount and sectioned inner ears to reveal pillar cells and Deiters' cells (Figure 4A, B) [29]. We found that in regions of the cochlea where outer hair cells are missing, a row of Prox1^+^ supporting cells are typically missing as well. Based on morphology and location, the missing supporting cell is likely a Deiters' cell or the outer pillar cell. In addition, we examined expression of the low affinity NGF receptor p75 that marks pillar cell apical processes (Figure 4C) and *β-tectorin*, which marks pillar cells and the greater epithelial ridge (Figure 4D). We found that in the mid-regions of the mutant cochlea individual pillar cell pairs are often absent, and replaced with an ectopic cell that is either a hair cell or an undifferentiated cell. *β-tectorin* is also absent in some regions of the mutant cochlea in a location corresponding to pillar cells, suggesting that the supporting cell loss is due at least in part to missing pillar cells. We also found that the hair cell marker parvalbumin is occasionally ectopically expressed in the Hensen's cell region of *Tg/Tg* mutants (Figure 4B). These ectopic parvalbumin-positive cells did not express other hair cell markers such as Myosin VI or show hair bundles with phalloidin staining, suggesting these ectopic cells are not *bona fide* hair cells, but rather represent mis-expression of at least one hair cell marker.

To determine if the cochlear defects we observed in *Tg/Tg* mice were due to gross abnormalities in the formation of the prosensory domain which gives rise to the organ of Corti, we examined mutant and wild-type animals for expression of the prosensory domain markers Sox2, and p27^kip1^ by antibody staining, and *Hey2* by in situ hybridization. We used Jag1 antibodies and an *in situ* probe for *Bmp4* to reveal the greater epithelial ridge and outer sulcus, respectively, which form a boundary with the prosensory domain. We did not observe any significant differences in the size of the prosensory domain, nor of the regions of the cochlea that border the prosensory domain in *Sfswap^Tg/Tg^* (Figure S2).

### *Sfswap^Tg/Tg^* mutant mice have severe vestibular organ defects

**Figure 5:**
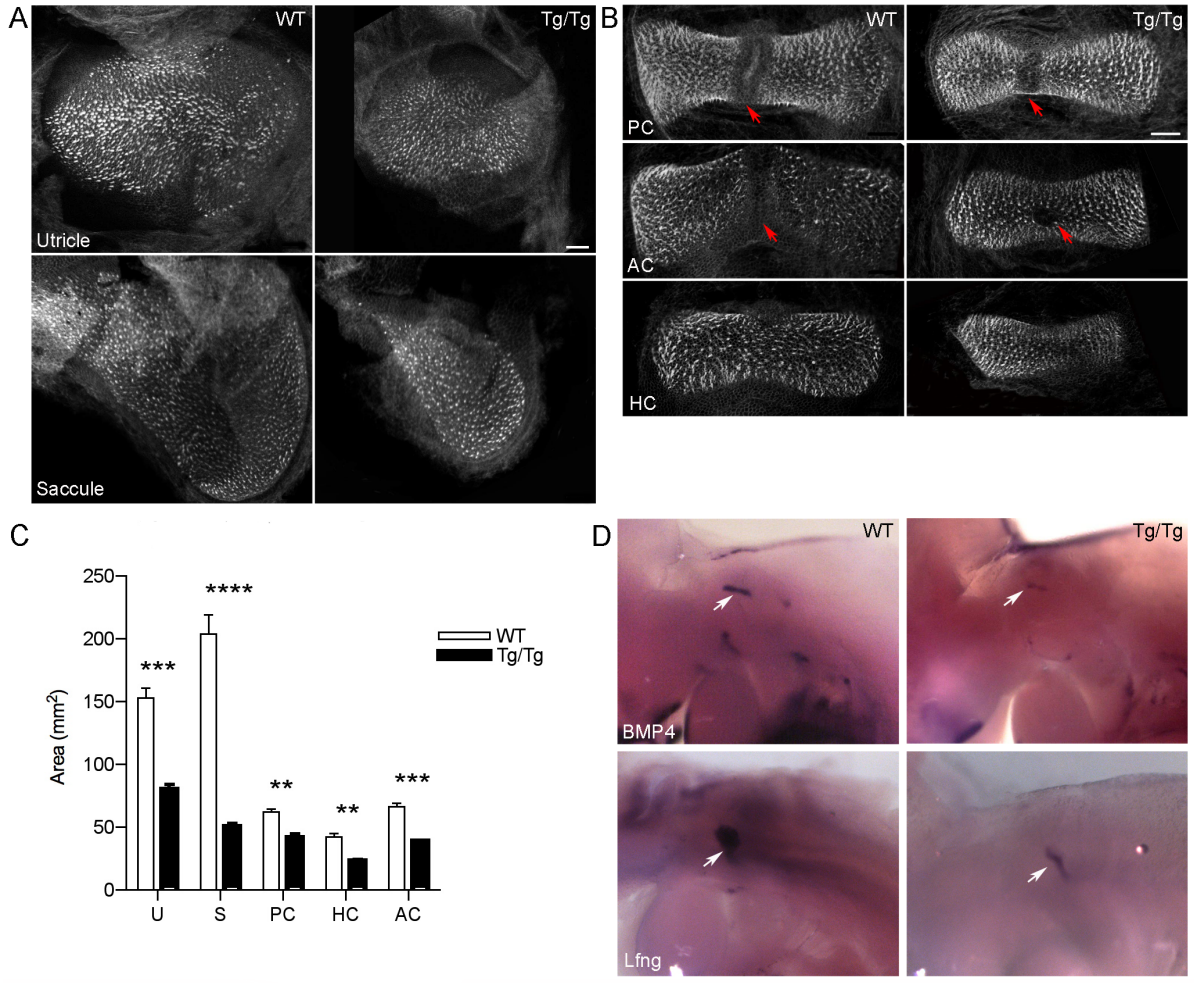
Mutation of *Sfswap* causes smaller vestibular cristae and maculae and misplaced eminentia cruciata. (A, B): Flat mount preparations of utricles and saccules and cristae from P0 *Sfswap^Tg/Tg^* and wild-type mice stained with fluorescently-labeled phalloidin to reveal hair cells. In addition to reductions in size (C), anterior semicircular canal eminentia cruciata are reduced in 85% of ears examined (B, red arrows denote eminentia cruciata). U: Utricle, S: Saccule, AC: Anterior crista, PC: Posterior crista, HC: Horizontal crista. Scale bars = 50 µm. Significant p-values are denoted with asterisks ** : p≤10^−3^, *** : p≤10^−4^, **** : p≤10^−5^ (D): Sensory patch primordia are reduced in size as revealed by *in situ* hybridization for *Bmp4* and *Lfng*.

As described above, *Sfswap^Tg/Tg^* mice were initially identified as exhibiting circling behavior indicative of a vestibular defect. To analyze cellular defects that might cause this behavior, we stained flat mounts of the cristae and maculae of newborn mice with fluorescently labeled phalloidin to detect actin in stereociliary bundles, and measured the area of the sensory structures. Anterior and horizontal canal cristae remained attached to the utricle during dissection so that they could be unambiguously identified based on location. All semicircular canal cristae and the maculae of the utricle and saccule are smaller in *Tg/Tg* mutants than in wild-type mice (Figure 5A, B, C). Strikingly, the saccule is reduced to 25% of wild-type size. In addition, the anterior semicircular canal cristae exhibited smaller or absent eminentia cruciata in 85% of ears examined. The defects observed in vestibular organs likely contribute to the circling behavior of *Sfswap* mutant mice. We further analyzed the source of this reduction by examining early known markers of the vestibular cristae and maculae, *Bmp4* and *Lunatic Fringe* (*Lfng*), respectively [30]. We found that the anterior stripe of *Bmp4* expression, which corresponds to the presumptive horizontal and anterior canal cristae, is reduced in mutants at E10.5. The posterior spot, which will develop into the posterior crista, is small or indiscernible at this age (Figure 5D). Similarly, *Lfng* expression, which marks the future utricle, saccule, and neurogenic domain, is severely reduced in mutants at E10.5 (Figure 5D). These data suggest that the size reduction in the maculae and cristae in *Sfswap^Tg/Tg^* mice may represent an early developmental defect.

Finally, we tested for defects in mechanotransduction in the hair cells of *Sfswap^Tg/Tg^* mutant pups by intraperitoneal injection of the dye AM1-43, which can be taken up through mechanotransduction channels [31]. We found no differences in AM1-43 uptake in maculae, cristae, or cochleas between mutant and control mice (Figure S3). This data combined with the ABR and DPOAE data suggest that the remaining hair cells in *Sfswap^Tg/Tg^* mutant mice are likely to be functional.

### *Sfswap* interacts genetically with *Jag1*

**Figure 6:**
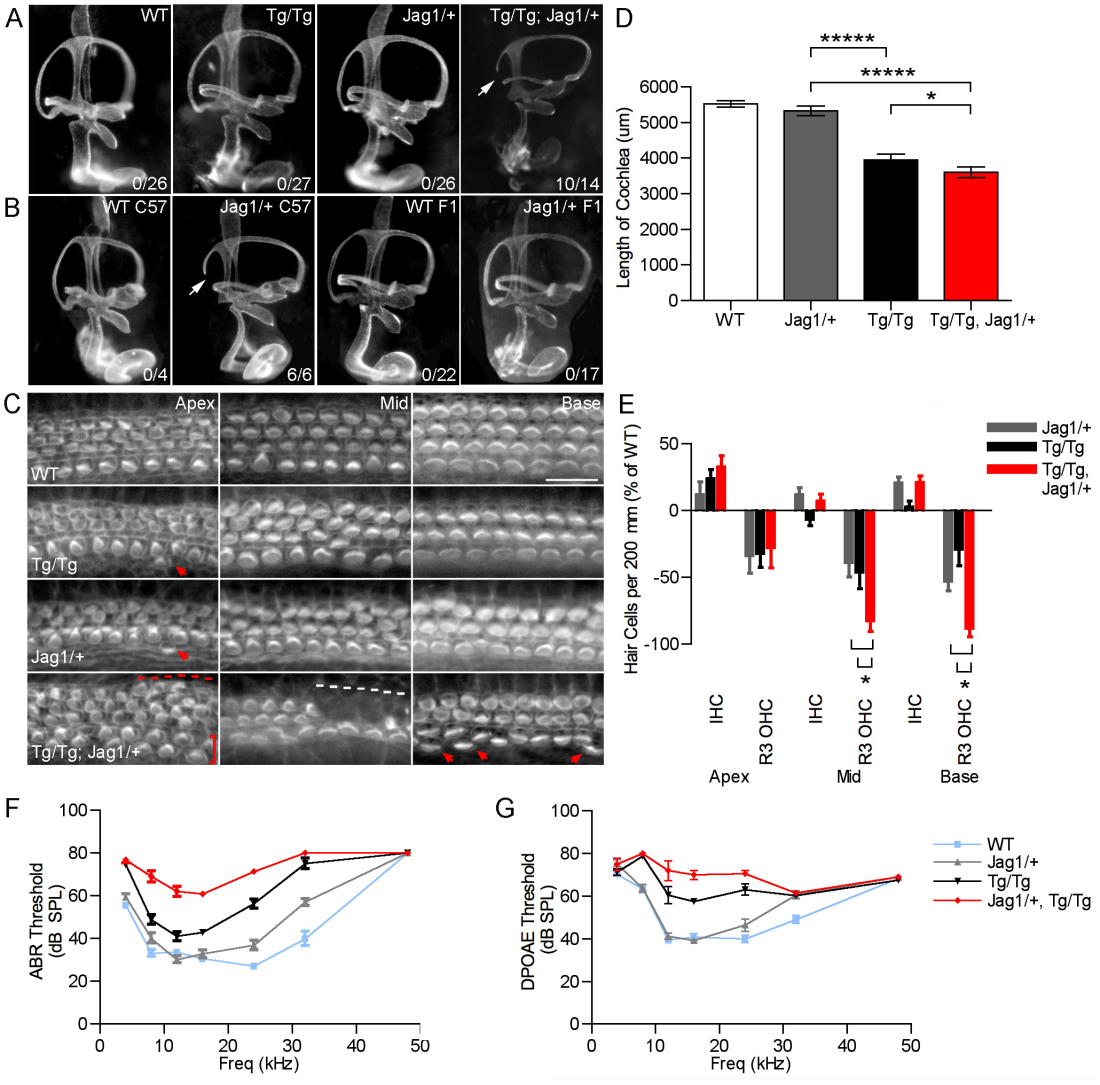
*Sfswap* interacts genetically with *Jagged1*. (A, B): Paint fills of E15.5 wild-type, *Sfswap^Tg/Tg^*, compound mutant (*Sfswap^Tg/Tg^, Jag1^+/−^*), and *Jag1^+/−^* inner ears. In the mixed FVBN/C57BL6 F2 background, semicircular canal truncations are not observed in *Sfswap^Tg/Tg^* or *Jag1^+/−^* mice. However, in *Sfswap^Tg/Tg^, Jag1^+/−^* mice, canal truncations are observed in 10/14 ears (arrow). Canal truncations are observed in *Jag1^−/+^* mutants on the C57BL6 background (B). However, when crossed one generation to FVB, this phenotype is suppressed (B, F1). The numbers in each panel refer to the number of ears with canal truncations. (C): An enhancement of hair cell phenotypes is observed in *Sfswap^Tg/Tg^, Jag1^+/−^* mutants compared to either *Sfswap^Tg/Tg^* or *Jag1^+/−^* mice. These enhanced defects include more ectopic inner hair cells in the base and mid-turn regions (arrowheads and brackets), loss of second and third rows of outer hair cells (white dashed line), and a 4^th^ row of outer hair cells in the apex (red dashed line). Scale bars = 20 µm. (D): The length of the *Sfswap^Tg/Tg^, Jag1^+/−^* mutant cochlea is significantly shorter than wild-type, *Sfswap^Tg/Tg^* or *Jag1^+/−^* mice. Asterisks denote p-values defined in Table 2 (* : p≤0.05, ***** : p≤10^−6^). (E): *Sfswap^Tg/Tg^, Jag1^+/−^* mutants have significantly fewer third row outer hair cells than either *Jag1^−/+^* or *Sfswap^Tg/Tg^* mice in the mid-turn and basal regions. Hair cells were counted in 200 µm lengths, and the change in hair cell numbers in *Sfswap^Tg/Tg^*, *Sfswap^Tg/Tg^, Jag1^+/−^* or *Jag1^+/−^* cochleas is shown compared to wild-type controls. (F) *Jag1^+/−^* mutants have significantly increased ABR thresholds particularly at high frequencies. Furthermore, *Sfswap^Tg/Tg^, Jag1^+/−^* mutant ABR thresholds are significantly increased compared to either *Sfswap^Tg/Tg^* or *Jag^+/−^* mutants. (G) *Jag1^+/−^* mutants have increased DPOAE thresholds at high frequencies compared to WT. *Sfswap^Tg/Tg^, Jag1^+/−^* have increased DPOAE thresholds compared to either *Sfswap^Tg/Tg^* or *Jag^+/−^* mutants.

*Sfswap^Tg/Tg^* mutants have a cochlear phenotype that is strikingly similar to that of *Jag1* heterozygotes and point mutants. *Jag1* heterozygous mutants display loss of the third row of outer hair cells, ectopic or extra inner hair cells, reduced expression of *Bmp4* and *Lfng* at 10.5, disruptions in canal cristae and utricular macula, and small body size [14], [15], [32]. These defects can vary according to the genetic background [33]. We found that *Jag1/+* mutants also have fewer supporting cells in a pattern similar to *Sfswap^Tg/Tg^* mutants (data not shown). These similarities lead us to hypothesize that *Sfswap* and *Jag1* function in the same genetic pathway. To test this, we crossed *Jag1* knockout mice (*B6;129S-Jag1^tm1Grid^/J* referred to hereafter as *Jag1^+/−^*) maintained on a C57BL/6 background, with our *Sfswap^Tg/+^* mutants raised in an FVB/N background to obtain *Sfswap^Tg/+^; Jag1^+/−^* F1 progeny. These were then crossed again to *Sfswap^Tg/+^* mice on an FVB/N background to generate *Sfswap^Tg/Tg^*; *Jag1+/*− mutants. Most of these compound mutants (10/14) exhibited semicircular canal truncations, but neither *Sfswap^Tg/Tg^* nor *Jag1^+/−^* mutants have canal truncations on this genetic background (Figure 6A). *Jag1^+/−^* mutants are known to have variations in semicircular canal defects depending on the genetic background [33]. To test if this is the case for the FVB/N background on which our *Sfswap^Tg^* mice were maintained, *Jag1^+/−^* mice were crossed one and two generations to wild-type FVB/N mice. Surprisingly, one generation was enough to completely suppress the canal truncations (Figure 6B). This indicates that there is a strong suppressor of canal truncations in the FVB/N background, and the canal truncations found in *Sfswap^Tg/Tg^*; *Jag1^+/−^* mice are therefore strongly indicative of an interaction between these two genes. To further test this hypothesis, we stained cochlear flat mounts from single and compound mutants with fluorescently labeled phalloidin to visualize stereocilia. We found that *Sfswap^Tg/Tg^*; *Jag1^+/−^* mutants have a more pronounced cochlear phenotype than either single mutant alone, showing a loss of outer hair cells extending into the second and first rows, increased inner hair cells throughout the length of the cochlea and the addition of a fourth row of outer hair cells in the apex (Figure 6C). In addition, compound mutant cochleas are significantly shorter than either *Sfswap* or *Jag1* mutant cochleas (Figure 6D, Table 2). Quantitatively, the number of outer hair cells in the mid-turn and base of the cochlea are significantly fewer in the compound mutant than either mutant alone (Figure 6E, Table 2). For audiological measurements, we out-crossed the *Jag1^+/−^* mice to the FVB background for an additional three generations. These *Jag1^+/−^* mice were then crossed to *Sfswap^Tg/+^* mice to generate compound heterozygotes that were then intercrossed to produce *Sfswap^Tg/Tg^*; *Jag1^+/−^*, *Jag1^+/−^* and *Sfswap^Tg/Tg^* mice. ABR and DPOAE measurements of compound mutants also showed increased threshold compared to those of *Tg/Tg* or *Jag1* mutants (Figure 6F, G; Tables 3–6). Interestingly, we find that *Jag1^+/−^* mice have a significant increase compared to WT ABR and DPOAE thresholds; this difference is evident at high frequencies. However, compound mutants also have increased thresholds compared to Tg/Tg at frequencies below 20 kHz, a range that is only mildly affected in *Jag1^+/−^* mice. The defects found in *Sfswap^Tg/Tg^*; *Jag1^+/−^* cochlear hair cells, semicircular canals, and auditory responses are greater than expected for a simple additive effect, implying a synergistic relationship between *Jag1* and *Sfswap* in inner ear development.

**Table 2:** *Sfswap^Tg/Tg^, Jag1^+/−^* mutants have enhanced phenotypes in the cochlea.

	Length	Apex IHC	Apex OHC	Mid IHC	Mid OHC	Base IHC	Base OHC
***Wild-type***	5213+/−211	24+/−1.3	91+/−3.3	25+/−1.5	80+/−4.2	21+/−0.5	74+/−1.1
***Jag1^+/−^***	5115+/−119	27+/−1.1	79+/−4.6	26+/−0.5	66+/−4.5	25+/−0.6	61+/−3.3
***Tg/Tg***	3822+/−115	30+/−1.1	73.3+/−2.7	23+/−1.2	64+/−3.7	21+/−0.7	67+/−3.2
***Jag1^+/−^Tg/Tg***	3365+/−179	33+/−2.3	71+/−5.7	26+/−1.4	53+/−2.8	25+/−0.8	51+/−2.1
***P(WT; Jag1^+/−^)***	NS	NS	NS	NS	NS	0.004	0.03
***P(WT; Tg/Tg)***	5.5E-5	0.002	0.002	NS	0.02	NS	NS
***P(WT; Tg/Tg, Jag1^+/−^)***	5.6E-5	0.01	0.02	NS	0.0005	0.01	3.5E-5
***P(Tg/Tg; Jag1^+/−^)***	9.3E-7	0.03	NS	NS	NS	0.002	NS
***P(Jag1^+/−^; Jag1^+/−^, Tg/Tg)***	4.1E-7	0.03	NS	NS	0.05	NS	0.03
***P(Tg/Tg; Jag1^+/−^, Tg/Tg)***	0.05	NS	NS	NS	0.04	0.009	0.002

Measurements of cochlear lengths reveal that *Sfswap^Tg/Tg^, Jag1^+/−^* mutants are significantly shorter than *Sfswap^Tg/Tg^* or *Jag1^+/−^* mutants. Similarly, at the base and mid, *Sfswap^Tg/Tg^, Jag1^+/−^* mutants have significantly fewer outer hair cells (OHC) than *Sfswap^Tg/Tg^* or *Jag1^+/−^* mutants alone.

**Table 3:** ABR ANOVA with repeated measures comparisons.

(I) Genotype	(J) Genotype	Mean Difference (I-J)	Std. Error	Significance
***WT***	Tg/Tg; Jag1/+	−28.5357	1.63400	1.14E-21
	Jag1/+	−5.2063	1.25785	1.51E-04
	Tg/Tg	−16.9286	1.30037	7.29E-17
***Jag1/+***	Tg/Tg; Jag1/+	−23.3294	1.68171	7.30E-18
	Tg/Tg	−11.7222	1.35985	4.39E-11
	WT	5.2063	1.25785	1.51E-04
***Tg/Tg***	Tg/Tg; Jag1/+	−11.6071	1.71375	2.21E-08
	Jag1/+	11.7222	1.35985	4.39E-11
	WT	16.9286	1.30037	7.29E-17
***Tg/Tg; Jag1/+***	Jag1/+	23.3294	1.68171	7.30E-18
	Tg/Tg	11.6071	1.71375	2.21E-08
	WT	28.5357	1.63400	1.14E-21

ANOVA with repeated measures were calculated to compare ABRs between genotypes. Jag1/+ mutants have a significantly increased ABR compared to WT. Compound mutants have a significantly increased ABR threshold compared to *Jag1^+/−^* and *Sfswp^Tg/Tg^* mice.

**Table 4:** ABR p-values for individual frequencies.

		4 KHz	8 KHz	12 KHz	16 KHz	24 KHz	32 KHz	48 KHz
***WT***	***Tg/Tg***	4.09E-10	2.31E-05	6.75E-03	7.03E-08	1.23E-10	5.07E-07	NS
***WT***	***Jag/+***	0.029	0.024	NS	NS	5.10E-04	4.75E-04	NS
***WT***	***Tg/Tg; Jag1/+***	1.74E-08	3.69E-08	5.14E-08	1.13E-10	2.86E-12	1.02E-05	NS
***Tg/Tg***	***Jag1/+***	9.91E-07	0.023	9.16E-04	2.64E-04	1.30E-05	1.04E-05	NS
***Tg/Tg***	***Tg/Tg; Jag1/+***	NS	2.73E-04	1.28E-04	8.11E-07	6.45E-04	NS	NS
***Jag1/+***	***Tg/Tg; Jag1/+***	1.74E-08	3.69E-08	5.14E-08	1.13E-10	2.86E-12	1.02E-05	NS

ABR p-values were calculated in pairwise combinations to compare genotypes at individual frequencies using Student's t-test.

**Table 5:** DPOAE ANOVA with repeated measures comparisons.

(I) Genotype	(J) Genotype	Mean Difference (I-J)	Std. Error	Significance
***WT***	Tg/Tg; Jag1/+	−18.1099	1.64224	3.18E-15
	Jag1/+	−3.4908	1.14980	3.74E-03
	Tg/Tg	−12.5385	1.29064	2.80E-13
***Jag1/+***	Tg/Tg; Jag1/+	−14.6190	1.65826	6.70E-12
	Tg/Tg	−9.0476	1.31097	7.11E-09
	WT	3.4908	1.14980	3.74E-03
***Tg/Tg***	Tg/Tg; Jag1/+	−5.5714	1.75885	2.57E-03
	Jag1/+	9.0476	1.31097	7.11E-09
	WT	12.5385	1.29064	2.80E-13
***Tg/Tg; Jag1/+***	Jag1/+	14.6190	1.65826	6.70E-12
	Tg/Tg	5.5714	1.75885	2.57E-03
	WT	18.1099	1.64224	3.18E-15

ANOVA with repeated measures were calculated to compare DPOAE thresholds. *Jag1^+/−^* mice have thresholds that are significantly higher than WT. Compound mutant DPOAE thresholds are also significantly higher than *Jag1^+/−^* and *Sfswap^Tg/Tg^* mice.

**Table 6:** DPOAE p-values for individual frequencies.

		4 KHz	8 KHz	12 KHz	16 KHz	24 KHz	32 KHz	48 KHz
***WT***	***Tg/Tg***	NS	7.23E-06	5.67E-06	1.53E-06	2.88E-07	2.97E-04	NS
***WT***	***Jag/+***	0.018	NS	NS	NS	NS	2.35E-04	NS
***WT***	***Tg/Tg; Jag1/+***	NS	3.10E-04	2.33E-08	3.72E-07	1.16E-07	2.80E-03	NS
***Tg/Tg***	***Jag1/+***	NS	1.99E-06	7.16E-05	8.31E-10	7.87E-04	NS	NS
***Tg/Tg***	***Tg/Tg; Jag1/+***	NS	NS	NS	3.17E-04	NS	NS	NS
***Jag1/+***	***Tg/Tg; Jag1/+***	NS	3.10E-04	2.33E-08	3.72E-07	1.16E-07	2.80E-03	NS

p-values were calculated in pairwise combinations using Student's t-test to compare DPOAE at each frequency between genotypes.

**Figure 7:**
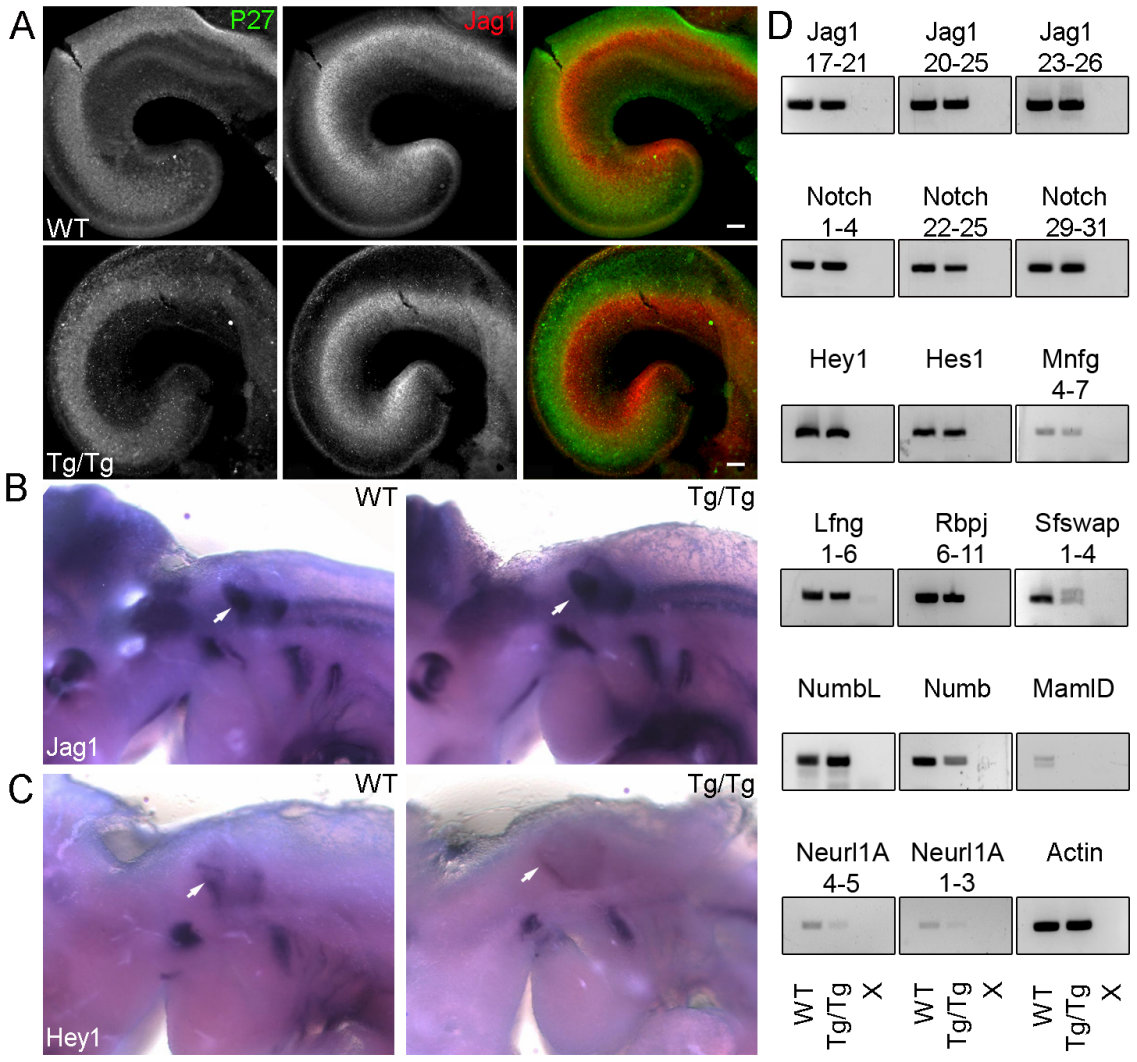
Jagged1 expression is not altered in *Sfswap^Tg/Tg^* ears but its downstream target Hey1 is reduced at E10.5. (A): Cochleas from E13.5 *Sfswap^Tg/Tg^* and wild-type mice co-stained with antibodies to p27^kip1^ (green) to reveal the prosensory domain and Jag1 (red) to show its expression in the adjacent Kölliker's organ. No significant difference is seen in Jag1 expression in the mutant cochlea. (B): Whole mount in situ hybridization of E10.5 *Sfswap^Tg/Tg^* and wild-type mice reveals no significant change in *Jag1* expression, but a significant down-regulation of its candidate downstream target, *Hey1* (C). Otocysts are denoted with white arrow. (D): cDNAs from E15.5 *Sfswap^Tg/Tg^* and wild-type inner ears were examined for splicing differences in Notch pathway genes by RT-PCR. No significant differences in splicing were detected in any gene except for *Sfswap*. The extra band in *Sfswap* mRNA in *Sfswap^Tg/Tg^* corresponds to 111 bp of transgene sequence that is spliced into the *Sfswap* mRNA exclusively in homozygous *Sfswap^Tg^* mice (data not shown). Levels of Neurl1 Numb mRNA were reduced, and MamlD1 expression was not detected. X refers to no cDNA controls.

To further analyze the mechanism for *Sfswap's* interaction with *Jag1*, we analyzed expression of Jag1 in cochlear whole mounts and at the otocyst stage, but found no differences in expression (Figure 7A, B). We next examined expression of the downstream target *Hey1* at E10.5 and found a significant reduction in expression (Figure 7C), despite normal *Jag1* expression. To determine if transcripts for genes in the Notch signaling pathway are incorrectly spliced in *Tg/Tg* mutants, we performed RT-PCR of splice junctions for Notch pathway genes using inner ear mRNA. We analyzed *Jag1*, *Notch1*, *Hes1*, *Hes5*, *Hey1*, *Hey2*, *HeyL*, *Mfng*, *Lfng*, *Rbpj*, *Delta1, Numb, NumbL, Maml1, Maml2, Maml3, MamlD1*, *Neuralized1A (Neurl1A)*, *and Sfswap* and found no differences in splicing in any gene except for *Sfswap* (Figure 7D and data not shown). We found slight reductions in levels of *Neurl1A* and *Numb* mRNA and significant reductions in *MamlD1* mRNA visible by RT-PCR, all of which could potentially contribute to the *Jagged1*-like phenotype of *Sfswap^Tg/Tg^* mice. To test for splice differences in these genes that would not be detectible by RT-PCR, we performed Northern blots using brain RNA. However, we found no detectable differences in splicing in *Sfswap* mutants in *Jag1*, *Neurl1A*, *MamlD1*, or *Numb* (Figure S4).

## Discussion

Using a lentivirus-based insertional mutagenesis strategy, we have identified the putative splicing factor Sfswap as an essential gene for inner ear development. *Sfswap^Tg/Tg^* mutants exhibit circling and balance dysfunction associated with vestibular defects and also have a moderate (20–25 dB) hearing loss. The mutants exhibit a partial loss of the third row of outer hair cells and show ectopic inner hair cells, together with some missing pillar cells. All organs in the vestibular system are smaller than their wild-type counterparts, particularly the utricle and saccule, which are less than 50% of the size of wild-type organs. Our data are consistent with *Sfswap* regulating aspects of Jagged1 signaling. First, the cochlear phenotype of *Sfswap^Tg/Tg^* mice is strikingly similar to that seen in *Jag1* heterozygous point mutants [14], [15]. Second, *Sfswap^Tg/Tg^* mice have otocyst patterning defects similar to those seen in *Jag1* mutants [32]. Finally, we show that our *Sfswap* mutant allele exacerbates the phenotype of *Jag1* heterozygous mice. We discuss these phenotypes in more detail below.

*Sfswap^Tg/Tg^* mutants have reduced levels of the early sensory markers *Bmp4, Lfng*, and *Hey1* and significantly smaller sensory organs. *Jag1* conditional mutants similarly have severely reduced vestibular structures [13]. *Jag1* mutants also show changes in expression of sensory markers such as *Bmp4, Sox2, Lfng and Hey1* at E10.25 [32]. Vestibular structures are also reduced in *Bmp4* conditional or hypomorphic mutants [34], particularly in the horizontal lateral canal. We suggest the reduction of *Bmp4* expression in our mutants may be due to defects in Jag1 signaling and leads to the smaller vestibular structures and reduced eminentia cruciata of the anterior crista that we observe in *Sfswap^Tg/Tg^* mutants.

The unique combination of loss of outer hair cells and ectopic inner hair cells that we observe in the cochleas of *Sfswap^Tg/Tg^* mutants has only been observed previously in heterozygous knockout or point mutants of *Jag1* [14], [15]. To date, there is no definitive molecular explanation for this unique phenotype. Kiernan and colleagues [14] suggested that Jag1 may have two roles, an early one in which Jag1 helps specify sensory organs, and a later one in which Jag1 helps define hair cell and supporting cell identity through lateral inhibition. We did not see significant changes in the size of the cochlear prosensory domain in *Sfswap^Tg/Tg^* mutants, although if there are small changes in the prosensory domain, they would be difficult to discern using the markers and techniques currently available. Ectopic rows of inner hair cells can be seen in *Jag1* heterozygotes and in apical regions of the *Jag1* conditional homozygote cochlea [6], [13]. In *Sfswap^Tg/Tg^* mutants, we also observe an extra row of inner hair cells in the apical region of the cochlea. Since Jag1 is expressed at the boundary of the greater epithelial ridge and the prosensory domain where the first inner hair cells form, it is possible that a partial loss of Jag1-Notch signaling leads to the inappropriate formation of extra inner hair cells in *Jag1* and *Sfswap^Tg/Tg^* mutants.

Although our data support a genetic interaction between *Sfswap* and *Jag1*, we currently have no evidence for a direct interaction. It is possible that *Jag1* mRNA splicing or stability is regulated by Sfswap. However, we did not detect significant differences in the levels or splicing pattern of *Jag1* transcripts, and we have not detected significant differences in Jag1 protein expression in the cochlea by immunofluorescence. Since the cochlear phenotype of *Jag1* mutants can vary according to both the type of mutation (point mutation versus null) and the genetic background on which mutants are maintained [13], [14], [15], [33], it is clear that hair cell patterning in the cochlea is exquisitely sensitive to levels of Jag1. It is possible that *Sfswap^Tg/Tg^* mice are causing small changes in *Jag1* expression below the limits of detection that are sufficient to affect cochlear patterning. Alternatively, it is possible that *Sfswap* is regulating the splicing or stability of modifiers of the *Jag1* locus. We screened a variety of Notch pathway genes for possible expression changes in *Sfswap* mutant cochleas. Several genes showed significant changes by RT-PCR including the mouse *neuralized* homologue *Neurl1A*, the Notch transcriptional co-activator *MamlD1*, and *Numb* (Figure 7D). Neurl1A is an E3 ubiquitin ligase that has been shown to regulate turnover and endocytosis of Jag1 *in vitro* [35]. It is therefore possible that small changes in Neurl1A activity may be sufficient to disrupt hair cell patterning. Similarly, Numb has been shown to regulate Notch signaling through endocytosis and ubiquitinization of the intracellular domain of Notch [36], [37]. Interestingly, Numb has recently been shown to be broadly expressed in the rat cochlea during development and over-expression can modulate levels of *Atoh1*, suggesting a possible role in hair cell development [38]. Mastermind proteins are transcriptional co-activators that are essential for Notch signaling [39]. It is possible that a reduction in *MamlD1* can affect Jagged1-Notch signaling through a reduction in canonical or non-canonical Notch signaling [40], [41]. It is also possible that the simultaneous reduction in two or more of these proteins results in the phenotypes seen in the *Sfswap* mutants.

*Sfswap* was discovered in *Drosophila* as a suppressor of the transposon-induced *white-apricot* mutation [16]. *White-apricot* mutants have a transposon insertion that disrupts the *white* transcript by splice inclusion and this disruption is partially suppressed by mutation of *Sfswap* through selective exclusion of the transposon [42], [43], [44]. Beyond this system, no evidence has been identified for the *in vivo* function or targets of *Sfswap* in *Drosophila*. In mice, *in vitro* studies have identified *Fibronectin* and *CD45* as putative splice targets of *Sfswap* [21], although *in vivo* targets have yet to be confirmed. In humans, the nonsyndromic autosomal dominant deafness locus *DFNA41* contains at least 100 genes, including *SFSWAP* [45]. The pathological mutation in this region has yet to be identified, making *SFSWAP* a potential candidate for future gene sequencing efforts.

Many genes undergo complex splicing patterns in the inner ear. These include several genes that are involved in ion transport including *Atp2b* [46], *Kcnq4* [47], [48], *Ca_v_1.3* [49], BK channels [50], [51], [52], and *P2X2* [53]. Spliced isoforms of these channels have been proposed to be involved in electrical differences in tonotopy, electromotility of outer hair cells, and differences between sensory and neuronal cells. Alternative splicing has been found to affect protein targeting [49], [54], [55], electrical properties channels [56], [57], [58], [59], cell viability [60], [61], and stereociliary organization [62] in the inner ear. There are also several examples of mutations in distinct alternative spliced isoforms that cause different pathologies in the ear. For example, loss of one isoform of *PCDH15* results in stereociliary defects, while mutations in the other two isoforms of this gene have no significant effect on hair cells [62]. Similarly, mutations in *harmonin* are typically associated with Usher Syndrome 1C, whereas a mutation in an alternative exon results in non-syndromic deafness at the DFNB18 locus with normal vision [63]. Despite the significance of alternative splicing in the inner ear, only one splicing factor has been identified that functions in the inner ear [61]. *Srrm4* is an SR-like protein that has recently been identified to be mutated in the Bronx waltzer (*bv*) mouse. Mutation at this locus results in degeneration of inner hair cells after E17.5 [64]. Gene ontology analysis of exons regulated by *Srrm4* suggests this factor regulates splicing of genes involved in synaptic transmission and the secretory pathway. We have now identified a second SR-like protein, Sfswap, which has distinct and highly specific effects on patterning of the inner ear. Our work provides the first evidence that a putative splicing factor is necessary for establishing the size of sensory organs in the ear and for proper patterning of mechanosensory hair cells in the organ of Corti.

## Materials and Methods

### Generation of *Sfswap* transgenic mice

A detailed account of the generation of *Sfswap* transgenic mice by lentiviral insertional mutagenesis with a tyrosinase minigene is given in Text S1.

### RNA analysis

For RT-PCR, RNA was isolated from E15.5 inner ears using the Ambion PureLink RNA mini kit (12183018A) according to the manufacturer's instructions. cDNA was generated using the Superscript III First Strand system (Invitrogen 18080-051) according to manufacturer's instructions. RT-PCRs for exon splicing events were performed using exon tiling primers (Tables S1 and S2). See Text S1 for Northern blot protocol.

### Probe generation

Plasmid probes for *in situ* hybridizations and Northern blots were obtained from Brigid Hogan (*Bmp4*),Gerry Weinmaster (*Jag1*), Elias Pavlopoulos (*Neurl1A*), Yutaka Hata (Numb, Addgene Plasmid 37012 [65]) or cloned using TOPO 2.1 vector (Life Technologies). PCR primers used to clone probes are as follows: *Sfswap* exons 1–4: F-GCTGTGTTGAAGTTGCGAAG and, R-CATCAGACGGGACGCTTAAT; *Sfswap* exons 15–18: F-AAAGGACCCGTTCCAGAAGT and R-CCACTGACTGACCCAGGAGT; *beta-Actin*: F- TGTTACCAACTGGGACGACA and R- AAGGAAGGCTGGAAAAGAGC; *MamlD1* F-TCCATTTCCCATCTCCTCAG and R- AGCCTTCCAAAAGCTCTTCC.

Some *in situ* probe templates were generated through direct PCR with the addition of a T7 promoter (T7 sequence is underlined). These include *Lfng*: F-GTTCCGCTCTGTCCATTGC R- GGATCCTAATACGACTCACTATAGGGAG CCCACTATGGGCGACTTTC and *Hey1*: F-AGACCTTGGGGGACAGAGAT and R- GGATCCTAATACGACTCACTATAGGGAGAACGGTGAAATCCGTGAGAC.

### Histology and *in situ* hybridization

For cryosections, heads were fixed in 4% paraformaldehyde overnight at 4 degrees. Heads were then washed briefly in PBS, immersed in 30% sucrose overnight, equilibrated in OCT, then embedded in OCT. Sections were taken between 8 and 14 µm. For P0 and older whole mounts, heads were fixed overnight in 4% paraformaldehyde. Cochleas, cristae, and maculae were then dissected out and processed. For immunohistochemistry, some tissue was first boiled for 10 minutes in 10 mM citric acid for antigen retrieval. All samples were washed 3 times in PBS, then 30 minutes in blocking buffer (PBS with 10% goat serum and 0.1% Triton-X or Tween-20). Tissue was then stained overnight in primary antibody diluted in blocking buffer. Tissue was then washed 3 times in PBS then incubated for 2 hours with the appropriate secondary antibody at 1∶400 dilution in blocking buffer. In tissues where Topro3 (1∶10,000, Invitrogen) or phalloidin (1∶200, Invitrogen) were used, they were added to the secondary antibody cocktail. Tissue was then washed 3 times in PBS and mounted in either Vectashield (Vector Labs) or Prolong Gold+Antifade (Invitrogen).

Antibodies used: Myo6 (Proteus), Prox1 (Millipore Bioscience Research Reagents), Parv (Sigma), Jag1 (Santa Cruz Biotechnology), p27^Kip1^ (Neomarker), p75 (Advanced Targeting Systems), Alexa 488 anti mouse and rabbit (Invitrogen), Cy5 anti mouse and rabbit (Jackson Immunolabs). *In situ* hybridization was performed as previously described [66], [67]. Digoxigenin labeled riboprobes were synthesized according to standard protocols [68].

### Imaging and measurements

Images were taken using a Zeiss LSM 510 confocal microscope, a Zeiss Axiophot microscope, or a Zeiss dissecting scope. Images were processed using Axiovision software or ImageJ, then further processed in Adobe Photoshop. Inner and outer hair cells were counted in P0 cochleas per 200 µm using Axiovision software. Cochlea lengths were measured at P0 using Axiovision software. Vestibular areas were measured on P0 vestibular flat mounts using ImageJ.

### Paint fills

Paint fills were performed as previously described [69], [70]. In brief, E13.5–E16.5 heads were fixed in Bodian's fix. Heads were dehydrated in an ethanol series, and then cleared with methyl salicylate. Inner ears were filled with white gloss paint in methyl salicylate using a Picospritzer III pressure injector (General Valve Corporation) and stored and photographed in methyl salicylate.

### Behavioral analysis and audiological measurements

Open field activity was measured using the Versamax System for automated activity recording. Mice were acclimated for at least 30 minutes to the testing room illuminated to 400 lux light and with 60 dB white noise before testing. The open field is a 40×40×30 cm Plexiglas arena. Tests were performed for 30 minutes during which time horizontal and vertical activity were measured via beam breaks. Chambers were cleaned with alcohol before and after each run to minimize interfering odors. Pre-pulse inhibition and startle responses were measured using a San Diego Instruments system. Mice were acclimated for at least 30 minutes in a separate nearby room before testing. Mice were placed in cylindrical restraint tube in a sound-attenuating chamber to minimize movement of the mouse and interfering noise. Mice were acclimated in the testing chamber to 70 dB white noise for 5 minutes before test pulses were delivered. Mice were then presented with 6 rounds of 8 test types in a pseudorandom order with 10–20 seconds between trials. These consist of no stimulus, startle at 120 dB for 40 ms, pre-pulse trials (74, 78, and 82 dB for 20 ms) and pre-pulse inhibition trials of each sound 100 ms prior to a 120 dB startle. Responses were recorded every 1 ms for 65 ms following the stimulus. *Tg/Tg* mice fail to present a threshold level startle response, so percent pre-pulse inhibition could not be calculated and only startle responses are reported. ABR and DPOAE were performed as previously described [71].

### Statistics

Significance was measured using students T-Test or ANOVA with repeated measures where appropriate using SPSS software. Graphs were generated using Prism.

### Ethics statement

All animal experiments in this study were carried out in accordance with the Institutional Animal Care and Use Committee at Baylor College of Medicine.

## Supporting Information

Figure S1 *Sfswap^Tg/Tg^* ABR traces are reduced compared to WT. Representative ABR traces are shown for *WT* and *Sfswap^Tg/Tg^* mice. *Tg/Tg* traces are qualitatively normal but have reduced peak-to-peak amplitude.
(TIF)

Figure S2 *Sfswap^Tg/Tg^* mutants show no distinctive changes in prosensory or nonsensory cochlear markers at E13.5. (A, B): Sections from E13.5 *Sfswap^Tg/Tg^* and wild-type mice co-stained with antibodies to p27^kip1^ (green) and Sox2 (red) to reveal the prosensory domain and Jag1 (red) to show its expression in the adjacent Kölliker's organ. No significant expression differences are seen in the mutant cochleas. (C): In situ hybridization of E13.5 *Sfswap^Tg/Tg^* and wild-type mice with the prosensory marker *Hey2* and the outer sulcus marker *Bmp4*. No significant differences are seen in the size or intensity of either domain.
(TIF)

Figure S3 *Sfswap^Tg/Tg^* mutants show no difference in hair cell mechanotransduction. Sfswap^Tg/Tg^ and wild-type pups were injected with AM1-43 at postnatal day 1 and examined at P2. Flat mount preparations of the vestibular organs (A) and cochlea (B) showed no significant differences in AM1-43 uptake.
(TIF)

Figure S4 *Sfswap^Tg/Tg^* mutants do not have splicing defects in *Jag1*, *Neurl1A*, *Mamld1*, or *Numb*. Northern blots were performed to examine splicing in putative *Sfswap* targets using brain RNA. No splicing differences were found in *Jag1*, *Neurl1A*, *Mamld1*, or *Numb*.
(TIF)

Table S1 List of RT-PCR primers for *Notch1, Delta1* and *Jagged1* exons.
(PDF)

Table S2 List of RT-PCR primers for Notch pathway targets and modifiers.
(PDF)

Text S1 This section contains a detailed account of the generation of *Sfswap* insertional mutant mice, together with a description of Northern blot methods.
(PDF)

Video S1 *Sfswap^Tg/Tg^* mutants exhibit circling and head bobbing behavior. *Sfswap^Tg/Tg^* mutants examined in their home cage exhibit significant circling behavior. Between circling bouts, *Sfswap^Tg/Tg^* mice show head bobbing behavior.
(MOV)
