# Supplementary figures and images for: The Candidate Splicing Factor Sfswap Regulates Growth and Patterning of Inner Ear Sensory Organs
Source: PLoS Genet. 2014 Jan 2;10(1):e1004055. doi: 10.1371/journal.pgen.1004055 (PMC3879212; doi:10.1371/journal.pgen.1004055)

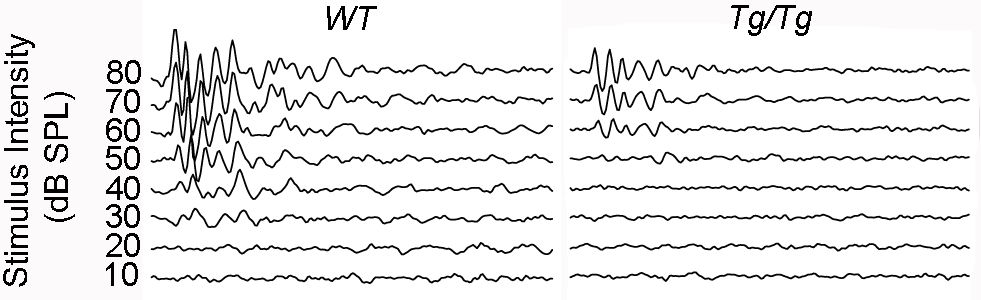

Supplement: Figure S1 — SfswapTg/Tg ABR traces are reduced compared to WT. Representative ABR traces are shown for WT and SfswapTg/Tg mice. Tg/Tg traces are qualitatively normal but have reduced peak-to-peak amplitude. (TIF) [file pgen.1004055.s001.tif]

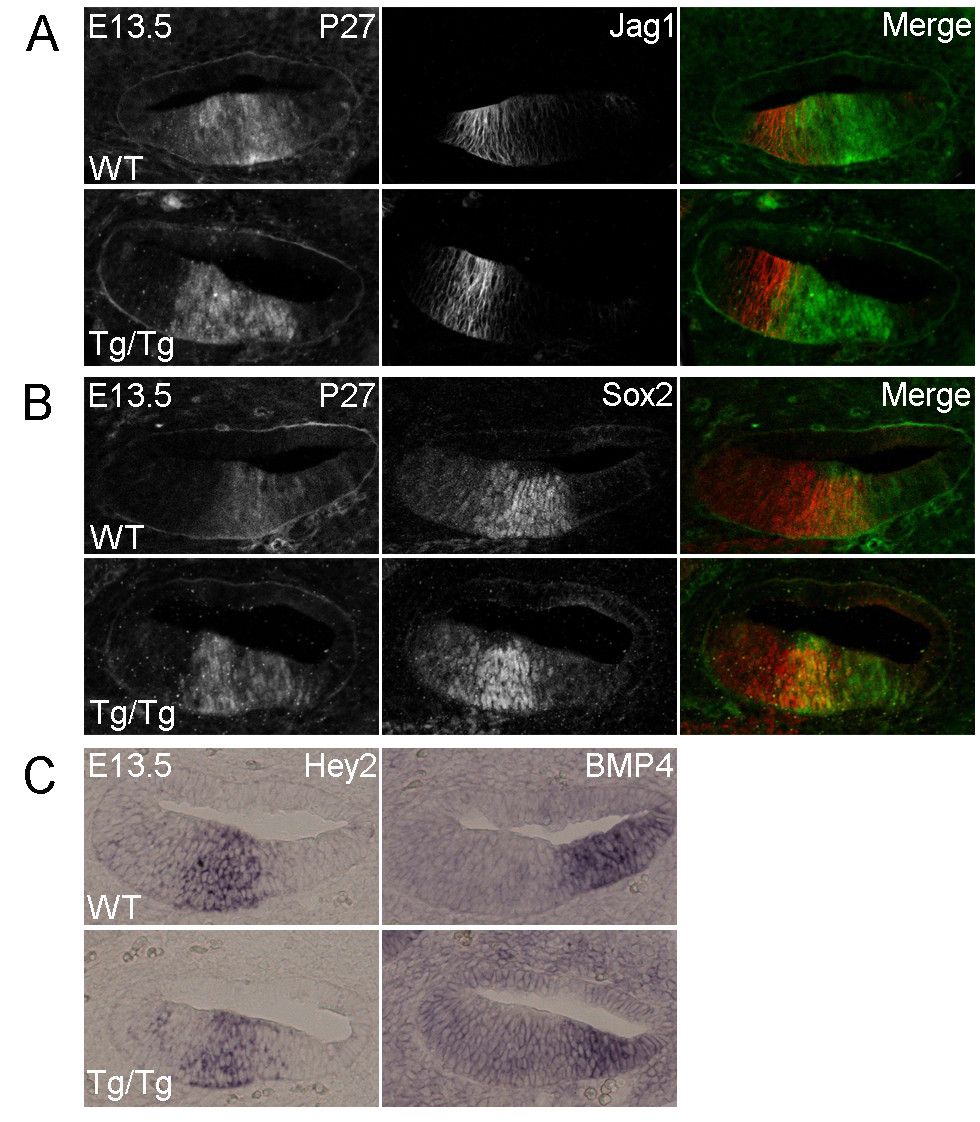

Supplement: Figure S2 — SfswapTg/Tg mutants show no distinctive changes in prosensory or nonsensory cochlear markers at E13.5. (A, B): Sections from E13.5 SfswapTg/Tg and wild-type mice co-stained with antibodies to p27kip1 (green) and Sox2 (red) to reveal the prosensory domain and Jag1 (red) to show its expression in the adjacent Kölliker's organ. No significant expression differences are seen in the mutant cochleas. (C): In situ hybridization of E13.5 SfswapTg/Tg and wild-type mice with the prosensory marker Hey2 and the outer sulcus marker Bmp4. No significant differences are seen in the size or intensity of either domain. (TIF) [file pgen.1004055.s002.tif]

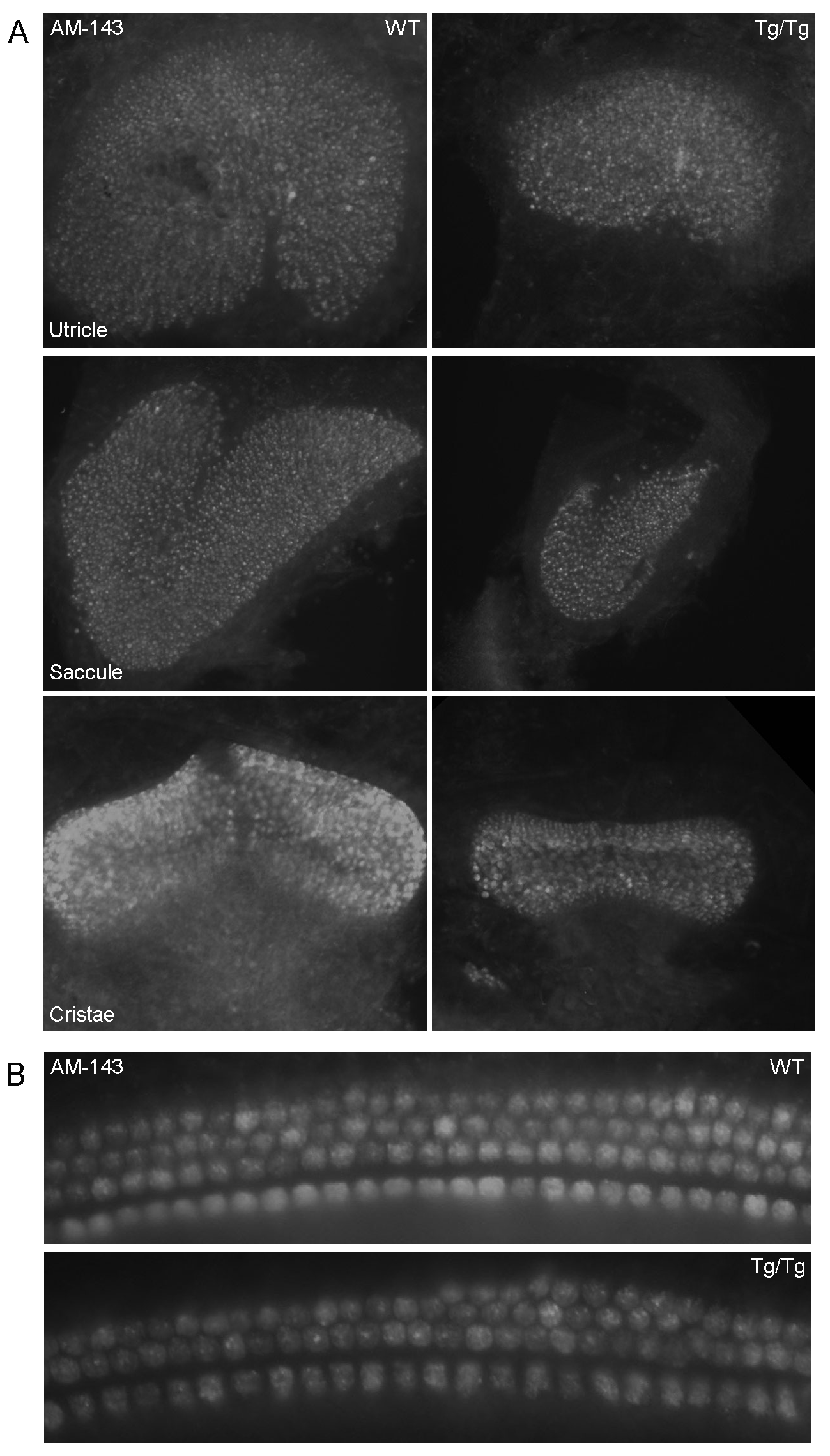

Supplement: Figure S3 — SfswapTg/Tg mutants show no difference in hair cell mechanotransduction. SfswapTg/Tg and wild-type pups were injected with AM1-43 at postnatal day 1 and examined at P2. Flat mount preparations of the vestibular organs (A) and cochlea (B) showed no significant differences in AM1-43 uptake. (TIF) [file pgen.1004055.s003.tif]

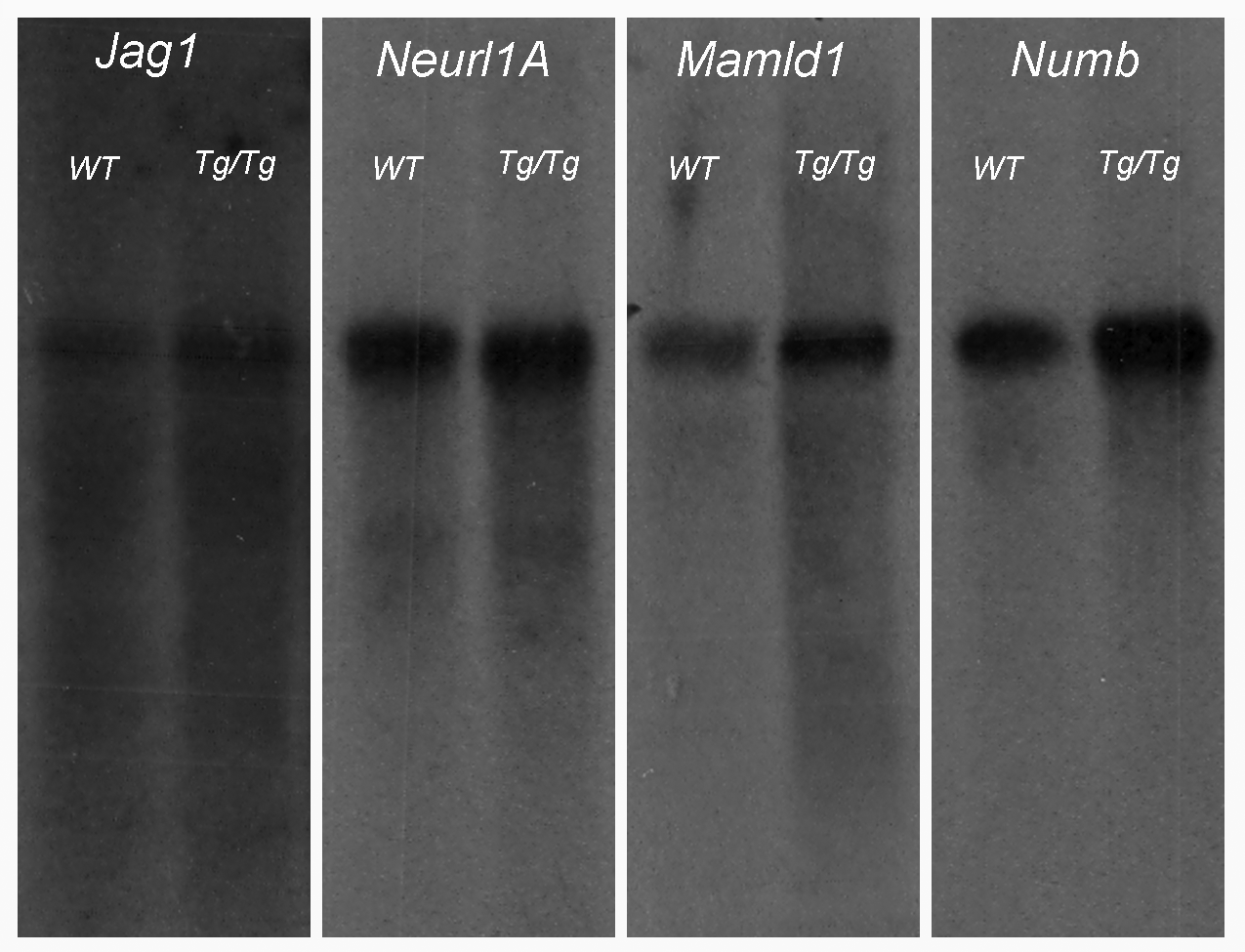

Supplement: Figure S4 — SfswapTg/Tg mutants do not have splicing defects in Jag1, Neurl1A, Mamld1, or Numb. Northern blots were performed to examine splicing in putative Sfswap targets using brain RNA. No splicing differences were found in Jag1, Neurl1A, Mamld1, or Numb. (TIF) [file pgen.1004055.s004.tif]
