# Supplementary material for: The Candidate Splicing Factor Sfswap Regulates Growth and Patterning of Inner Ear Sensory Organs
Source: PLoS Genet. 2014 Jan 2;10(1):e1004055. doi: 10.1371/journal.pgen.1004055 (PMC3879212; doi:10.1371/journal.pgen.1004055)
Supplement: Table S1 — List of RT-PCR primers for Notch1, Delta1 and Jagged1 exons. (PDF) [file pgen.1004055.s005.pdf]

Table S1. RT-PCR primers for *Notch1*, *Delta1*, and *Jagged1*

Gene	Exons Detected	Forward	Reverse
Sfswap	3-5	GCGGAGGATGGAAACTACAG	CTTGGCCTTCAGCATGATCT
Jag1	1-5	CCCCCTGAGTCTTCTGCTC	AGGGTTTATCATGCCTGAGTG
	3-7	GCCTGGGATTCCAGTAATGA	TGATGAGTCCCACAGTAATTCAG
	6-11	CAGTGCCTCTGTGAGACCAAC	AGGAGGCGTCATTCTGACACT
	10-14	CTGGATGGGTCAGAACTGTG	GATATACCGCACCCCTTCAG
	13-18	AAAGACCACTGCCGTACCAC	GGCAGGCAGCTACTGTTTCTA
	17-21	CTGGGAAGGAACAACCTGTAA	AGCCATTGATCTCATCCACAC
	20-25	GGGCCAGACTGCAGGATAA	CTTGACAGGGTTCCCATCAT
	23-26	GGTCCTGGATGACCAGTGTT	CCGCTTCCTTACACACCAGT
	25-26	GGGAACCCTGTCAAGGAAAT	GGGATGCTTCCAACTTCACA
Notch1	1-4	CAGGAAAGAGGGCATCAGAG	GGCAGCGACAGATGTATGAA
	8-13	CAGATTGGGGAGTTCCAATG	CAATGTTGACGTTACACATGCTT
	12-16	GCTACGAATGTGCCTGTGAA	TGTGGGACAGACACAGGAAA
	15-19	GACCTGCATTGATGATGTCG	CAGGTGGACACAGACAGGTG
	18-21	GCCAATTGCACTGACTGTGT	GGGTTAGGTGAGCACTCGTC
	20-23	GACGTGCTCAGTGTGTCCTG	ACACAGGTGCCATTGTTGAA
	22-25	GGGGTACCTGCATTGATCTG	TCACAGGTGCCCTGATTGTA
	24-26	CAAGAATGGGGGTGTCTGTG	TGGTCCTTGCAGTACTGGTC
	25-27	ACTGTGACAGCCAGTGCAAC	CGGCCTCAATCTTGTAAGGA
	26-30	ACTATGGCCACGAGGAAGAG	CAGGGAGAACTACTGGCTCCT
	29-31	AATGCCTCAGATGGTGCTCT	CACTGTTGCCTGTCTCAAGG
Delta1	1-4	TCCCTGGGTCTTTGAAGAAG	GTGCTCGTCACACACAAACC
	3-7	CAGACTCTCCCGATGACCTC	GGGCTAGGAGCACACTCATC
	6-9	TCCGATACCCAGGTTGTCTC	GGCAGGTACAGGAGAAGTCG
	9-11	CAACAAGAAGGCGGACTTTC	TCCTCTCTCAGCAGCATTCA
	8-10	TAACCCTGACGGAGGCTACA	TCTGGCCTTTTTCTGTCAGG
