# Supplementary material for: The Candidate Splicing Factor Sfswap Regulates Growth and Patterning of Inner Ear Sensory Organs
Source: PLoS Genet. 2014 Jan 2;10(1):e1004055. doi: 10.1371/journal.pgen.1004055 (PMC3879212; doi:10.1371/journal.pgen.1004055)
Supplement: Table S2 — List of RT-PCR primers for Notch pathway targets and modifiers. (PDF) [file pgen.1004055.s006.pdf]

Table S2. RT PCR primers for Notch signaling targets and modifiers.

Gene	Exons Detected	Forward	Reverse
Hey1	All	CCCAGACTACAGCTCCTCAGA	ATGCTCAGATAACGGGCAAC
Hey2	All	ATGAAGCGCCCTTGTGAG	AGCCACTTCTGTCAAGCACTC
Hes5	All	ATGCTCAGTCCCAAGGAGAA	AGCAGCTTCATCTGCGTGT
Hes1	All	AAACGAAAATGCCAGCTGAT	GTCACCTCGTTCATGCACTC
HeyL	All	TGTGGGTCAAGAGAACGATCTTA	CCCCAGGTATCTTATGACCTCAG
Lfng	1-6	CTGCTGTTCGAGACCTGGAT	CAATGTAGCCAATGGTGCAG
	4-8	AGCTATCCCCACACCCAAG	TACAGGTGGCAATGGACAGA
Mfng	All	GTCTCCAGGATCAGGCAACA	CTGGGTAGAGGAGACAATGGA
	processed transcript	GCTTTCCTGTCGTCTTGCTC	GCTCTGCAGAACAGTTGGTG
	4-7 (retained intron)	TTCACTGACAGCCCAGATGA	GCACCATCTTCAAAGCCAGT
	5-7 (retained intron)	GAACGCCTCCAGGAGAGACT	GGTGGCTAGGAGTTTGGTCA
Neurl1a	1-3 (201)	CAGATCACAAGGAACACTATCCA	CACAGGCTCACCGACAGG
	2-5	GACGTCTACGGCCTCACG	AGGTGAGTTGGGGGCTGT
	1-3 (202)	ACTTCTCCAGTGTCTCCTCTCTG	GCACAGGCTCACCGACAG
	1-2 (201)	GAAGGAGGAGGGGAACTGG	TTCAGCCTGACTTGCTCGTA
	2-3	GCTCTTCCATCCCCACACTA	GACCCCACTGAAGAAAAGCA
	3-4	AGCATTGCCTGAGGAGTTTG	GGCTGTGCTGAGAATTGAGC
	4-5	GAGCTGCACCTGAGTCACAA	GACTGTGTCCACTGCGTGTT
	1-2 (202)	GCACACACTTGCACACCTTT	GAGGATCTGGGAGCCCTTAG
MamlD1	3-6	TGATTCTATGCCTGCTCTGC	ATCTTTTGCTGGTTGGATGG
Maml2	All	TTATCAAGCCAGCCTTTGCT	TGAGGCTGGAGTTTGGAGTT
Maml3	2-5	AACGCATGCTTCTCATTAAGC	AGCCACCTGGTTTCTCACAG
Maml1	2-5	TCAGAACCCCGTGAATAACC	GAGGACAGCTGGAGTTGGAC
Numb	2-8	GGCGGAGGTAGAGTCTGAGG	GGCCTTCACTGCTTTCTTTC
	7-11	CAGTTGCAAGATGCCAAGAA	GAGAGGCAGCACCAGAAGAC
Numbl	1-6	CACAGCAGCAGCAGCACT	CAGGAGCACAGAAGGAGACC
	Retained intron	CACAGCAGCAGCAGCACT	TGGAATCGGCTCAGTTAAGG
Rbpj	1 (202)-4	ACTCGAGGCGTCTGCTTAAC	CCAACCACTGCCCATAAGAT
	1 (201)-4	GGACTACTCGGAGGGCTTGT	CCAACCACTGCCCATAAGAT
	1 (203)-4	TGGAGTTTTGGCGAGAGTTT	CCAACCACTGCCCATAAGAT
	3-7	AACGAGGGGATCAAACAGTG	CTGCCCGTAATGGATGTAGC
	6-11	GAATTTCCACGCCAGTTCAC	GCAGAAATGTCTGGGACCAC
