# Supplementary material for: The Candidate Splicing Factor Sfswap Regulates Growth and Patterning of Inner Ear Sensory Organs
Source: PLoS Genet. 2014 Jan 2;10(1):e1004055. doi: 10.1371/journal.pgen.1004055 (PMC3879212; doi:10.1371/journal.pgen.1004055)
Supplement: Text S1 — This section contains a detailed account of the generation of Sfswap insertional mutant mice, together with a description of Northern blot methods. (PDF) [file pgen.1004055.s007.pdf]

Supplementary Methods

Generation of Mice

Sfswap$^{Tg(Tyr)2267BOve}$ mice were generated by using a self-inactivating (SIN) lentivirus to generate transgenic mice with single-copy, integrase-mediated, insertion sites. The SIN-lentivirus was engineered to contain a tyrosinase minigene in place of the ubiquitin promoter and EGFP coding sequences in the FUGW lentivirus [1] The tyrosinase minigene contains a 600 bp mouse tyrosinase enhancer, a 600 bp mouse tyrosinase promoter, and the tyrosinase coding sequences. The virus contains a WPRE (woodchuck hepatitis virus pos-transcriptional regulatory element) downstream from the minigene. Expression of tyrosinase rescues albinism in mice. Transgenic mice were generated by subzonal injection of the packaged lentivirus into two-cell stage FVB/N embryos at a concentration of $1x10^8$ pfu/ml. The first day of infections produced 28 pigmented, chimeric, F0 mice. F0 mice were bred to FVB/N partners to generate non-chimeric F1 offspring. Most F0 mice produced F1 mice with different coat colors, indicating more than one integration site per founder. F1 mice were mated to FVB partners to establish families with single lentiviral insertion sites. These families were inbred to look for mutant phenotypes. The F0 mouse for family OVE2267 (a male) produced medium grey offspring (2267A) and tan offspring (2267B). Homozygous 2267B mice exhibited a behavioral phenotype**.** Inverse PCR was performed to identify the lentiviral integration site. 1-2µg of genomic DNA was digested with NsiI, the enzyme was deactivated, and then the genomic fragments were circularized by ligation using T4 ligase (NEB). DNA was then precipitated and resuspended in Tris buffer. Primers (Psi(AS)#1-ctcgcacccatctctctccttcta and Env(S)#1-cccgaaggaatagaagaagaaggtg) were used for the first round of amplification. A second round of amplification was performed using the nested primers (Psi(AS)#2-tcctctggtttccctttcgctttca and Env(S)#1-

ccattcgattagtgaacggatcgg). The second PCR reaction was run out on a 1% agarose gel and the PCR band was purified using the GE gel band purification kit (#28-9034-71). The purified band was sequenced using the sequencing primer 5'LTRAS#1-cctggtgtgtagctttgccaatca. The sequence was used for a Blat search to identify the exact insertion site. In the OVE2267B line, the lentivirus integrated in the sense orientation in the 4th intron of *Sfswap*. Mice were subsequently genotyped based on coat color or by direct PCR using the upstream primers OVE2267BChrLF-TAGCAGCAGATACCGTTTACCATA and the downstream primer OVE2267BChr5RF-TACTTTTCTTCTCCTCGTTCTTGC to amplify the wild-type chromosome or OVE2267BChrLF and primer 5'LTR caaggatatcttgtcttcgttggg to amplify the transgenic allele. For genetic interaction experiments, *Sfswap*$^{Tg/+}$ mice were crossed to *Jag1*^{tm1Grid} heterozygous mice obtained from Jackson Labs (strain # 010616). Jag1 mice were genotyped using primer sequences provided by Jackson labs. Double heterozygous mice were crossed to *Sfswap*$^{Tg/+}$ to obtain compound mutants.

Northern Blots

Northern blots were performed on RNA from adult brain. Total RNA was isolated using the Ambion PureLink RNA mini kit according to the manufacturer's instructions. DNA was removed using Turbo DNAse (Ambion). For some probes, polyA RNA was isolated using the NucleoTrap mRNA mini kit (Machery-Nagel). RNA was run overnight in a 1% formaldehyde gel and transferred to Hybond N+ membranes. Probes were labeled with αP32-dCTP using the GE Rediprime II kit and hybridized with in a solution containing 10% dextran sulfate, 1M NaCl, 0.05M Tris pH 7.5, 1% SDS, and 4 mg herring sperm DNA overnight. Membranes were washed in 0.1% SDS and 0.2x SSC and film was exposed between 6 hours and 1 week.

1. Lois C, Hong EJ, Pease S, Brown EJ, Baltimore D (2002) Germline transmission and tissue-specific expression of transgenes delivered by lentiviral vectors. Science 295: 868-872.
